# Aβ-dependent reduction of NCAM2-mediated synaptic adhesion contributes to synapse loss in Alzheimer's disease

**DOI:** 10.1038/ncomms9836

**Published:** 2015-11-27

**Authors:** Iryna Leshchyns'ka, Heng Tai Liew, Claire Shepherd, Glenda M. Halliday, Claire H. Stevens, Yazi D. Ke, Lars M. Ittner, Vladimir Sytnyk

**Affiliations:** 1 School of Biotechnology and Biomolecular Sciences, University of New South Wales, Sydney, New South Wales 2052, Australia; 2 Neuroscience Research Australia, Sydney, New South Wales 2031, Australia; 3 Dementia Research Unit, School of Medical Sciences, The University of New South Wales, Sydney, New South Wales 2052, Australia

## Abstract

Alzheimer's disease (AD) is characterized by synapse loss due to mechanisms that remain poorly understood. We show that the neural cell adhesion molecule 2 (NCAM2) is enriched in synapses in the human hippocampus. This enrichment is abolished in the hippocampus of AD patients and in brains of mice overexpressing the human amyloid-β (Aβ) precursor protein carrying the pathogenic Swedish mutation. Aβ binds to NCAM2 at the cell surface of cultured hippocampal neurons and induces removal of NCAM2 from synapses. In AD hippocampus, cleavage of the membrane proximal external region of NCAM2 is increased and soluble extracellular fragments of NCAM2 (NCAM2-ED) accumulate. Knockdown of NCAM2 expression or incubation with NCAM2-ED induces disassembly of GluR1-containing glutamatergic synapses in cultured hippocampal neurons. Aβ-dependent disassembly of GluR1-containing synapses is inhibited in neurons overexpressing a cleavage-resistant mutant of NCAM2. Our data indicate that Aβ-dependent disruption of NCAM2 functions in AD hippocampus contributes to synapse loss.

## Abstract (web summary)

Understanding how ß-amyloid contributes to synapse loss and dysfunction is a central goal of Alzheimer's disease research. Here, Leshchyns'ka *et al.* identify a novel mechanism by which Aß disassembles hippocampal glutamatergic synapses via cleavage of a neural cell adhesion molecule 2 (NCAM2).

## Graphical abstract

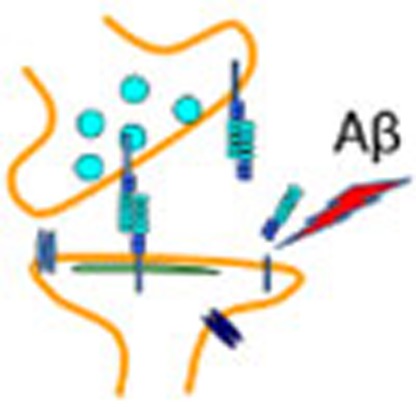

Learning and memory processes depend on the number and correct functioning of synapses in the brain. Cell adhesion molecules are enriched in the pre- and postsynaptic membranes. These molecules physically connect synaptic membranes, providing mechanical stabilization of synaptic contacts^1^,^2^,^3^, are necessary for the formation of new synapses during neuronal development^4^,^5^, and maintain and regulate synaptic plasticity in adults^6^,^7^,^8^,^9^,^10^.

Alzheimer's disease (AD) is a neurodegenerative brain condition predominantly of the aging population. One of the earliest signs of AD is the loss of synapses^11^, which can at least partially be linked to the toxicity mediated by Aβ^12^,^13^,^14^, a peptide that accumulates in the brains of AD patients. The impact of AD on synaptic adhesion and the role of synaptic cell adhesion molecules in the progression of the disease remains poorly understood.

The neural cell adhesion molecule 2 (NCAM2), sometimes designated OCAM, belongs to the immunoglobulin superfamily of cell adhesion molecules. NCAM2 participates in homophilic trans-interactions^15^,^16^. During human embryonic development, NCAM2 is expressed in several tissues, including lung, liver, and kidney with the highest expression in the brain^17^. The expression level of NCAM2 peaks around postnatal day 21 and remains high during adulthood^15^, suggesting that the protein is necessary both during development and in adult brains. Accordingly, studies with cultured neurons and in NCAM2 deficient mice show that NCAM2 is important for the development of the brain, and the olfactory system in particular^18^,^19^.

The *NCAM2* gene is located on chromosome 21 in humans and NCAM2 overexpression has been suggested to be one of the factors contributing to the symptoms of Down syndrome^17^, which presents with early-onset AD pathology. Single-nucleotide polymorphisms in the NCAM2 gene have been reported as a risk factor related to the progression of AD in the Japanese population^20^. A recent genome-wide association study has found an association between single-nucleotide polymorphisms in the *NCAM2* gene and levels of Aβ in the cerebrospinal fluid in humans, suggesting that NCAM2 is involved in the pathogenic pathway to the senile plaques that concentrate in AD brains^21^. Since genetic association studies indicate a link between NCAM2 and AD, we have analysed whether AD pathology influences levels of NCAM2 in synapses. Our results indicate that the synaptic adhesion mediated by NCAM2 is highly susceptible to Aβ toxicity and that proteolytic fragments of NCAM2 generated in an Aβ-dependent manner can directly contribute to the induction of synapse disassembly.

## Results

### Synaptic NCAM2 is reduced in the hippocampus in AD

To analyse whether functions of NCAM2 are affected in AD, frozen post-mortem brain tissue of AD patients and non-affected controls (*n*=10 each) was analysed by western blot with antibodies against NCAM2. The detailed demographic data for the subjects analysed are presented in Supplementary Table 1. Total levels of NCAM2 were slightly increased in the hippocampus, but not significantly affected in the cerebellum or superior temporal cortex in AD (Supplementary Fig. 1). In contrast, levels of VGLUT1, a presynaptic marker-protein of excitatory synapses, were reduced in AD hippocampus (Supplementary Fig. 1), indicating a loss of excitatory synapses. Levels of VGAT, a presynaptic marker-protein of inhibitory synapses, were not significantly affected in any brain region analysed (Supplementary Fig. 1).

**Figure 1:**
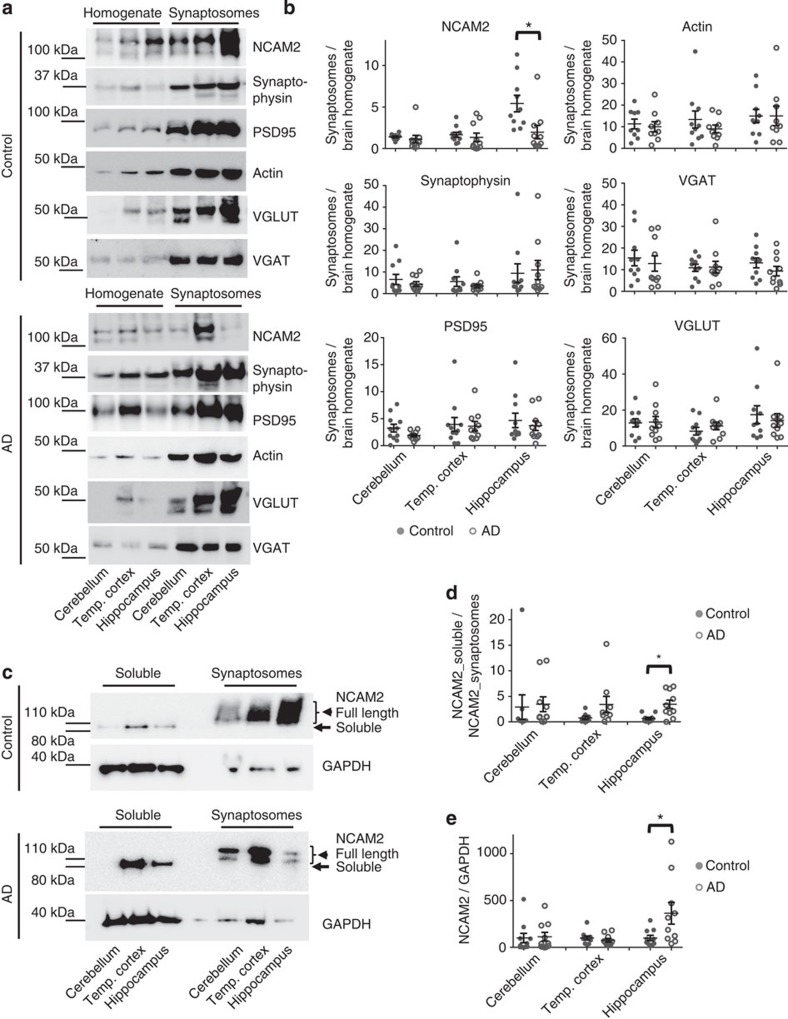
Synaptic accumulation of NCAM2 is reduced in the hippocampus of AD-affected individuals. (**a**) Western blot analysis of homogenates and synaptosomes prepared using brain tissue from cerebellum, temporal cortex and hippocampus of control and AD individuals. Note enrichment of synaptophysin, PSD95, actin, VGLUT and VGAT in synaptosomes indicating efficient synaptosome isolation. NCAM2 is highly enriched in synaptosomes versus homogenates from the hippocampus in control but not in AD individuals. Full-length versions of the western blots are shown in Supplementary Figs 6 and 7. (**b**) Graphs show the ratio of the respective protein levels in synaptosomes to homogenates for individual cases and mean±s.e.m. (*n*=10 control and *n*=10 AD cases were analysed). *P=0.0039, Mann–Whitney test. (**c**) Western blot analysis of the soluble protein fractions and synaptosomes prepared using brain tissue from the cerebellum, temporal cortex and hippocampus of control and AD individuals. Total protein concentration in synaptosomes was kept at 25% of that in the soluble protein fraction to improve visualization of the protein bands in both fractions on one blot. Probes were analysed with antibodies against the extracellular domain of NCAM2 and GAPDH, which served as a loading control. Note increased levels of soluble ∼100-kDa NCAM2 fragments in the soluble protein fraction from the hippocampus and temporal cortex of the AD individuals. Full-length versions of the western blots are shown in Supplementary Fig. 8. (**d**,**e**) Graphs show NCAM2 levels in the soluble protein fraction normalized to NCAM2 levels in synaptosomes (**d**) and NCAM2 levels in the soluble protein fraction normalized to GAPDH levels in homogenates (**e**) for individual cases and mean±s.e.m. (*n*=10 control and *n*=10 AD cases were analysed). *P<0.05, Mann–Whitney test.

Changes in the protein levels in brain homogenates do not necessarily reflect changes in the protein levels in synapses. To analyse whether the synaptic function of NCAM2 is affected in AD, we compared the enrichment of NCAM2 in synaptosomes isolated from the brain tissue of individuals with AD and non-affected controls by western blot analysis of synaptosomes and total homogenates of the brains used for synaptosome preparations. Equal total protein amounts from each probe were applied to the gels to compensate for any possible differences in the yield of synaptosomes because of the synapse loss observed in AD. Western blot analysis with antibodies against actin, VGLUT1, VGAT, synaptophysin (a general presynaptic marker-protein), and PSD95 (a postsynaptic marker-protein), showed that these proteins were enriched to similar levels in synaptosomes from AD and control brains, indicating similar purities of intact synaptosome isolations (Fig. 1a). Western blot analysis showed that in control individuals NCAM2 was highly enriched in synaptosomes from the hippocampus and to a lower degree in synaptosomes from the temporal cortex and cerebellum (Fig. 1a,b). This synaptic enrichment of NCAM2 was significantly reduced in synaptosomes from AD hippocampi (Fig. 1a,b). The synaptic enrichment of NCAM2 was slightly lower in the AD versus control cerebellum, however the difference was not statistically significant (Fig. 1a,b).

**Figure 2:**
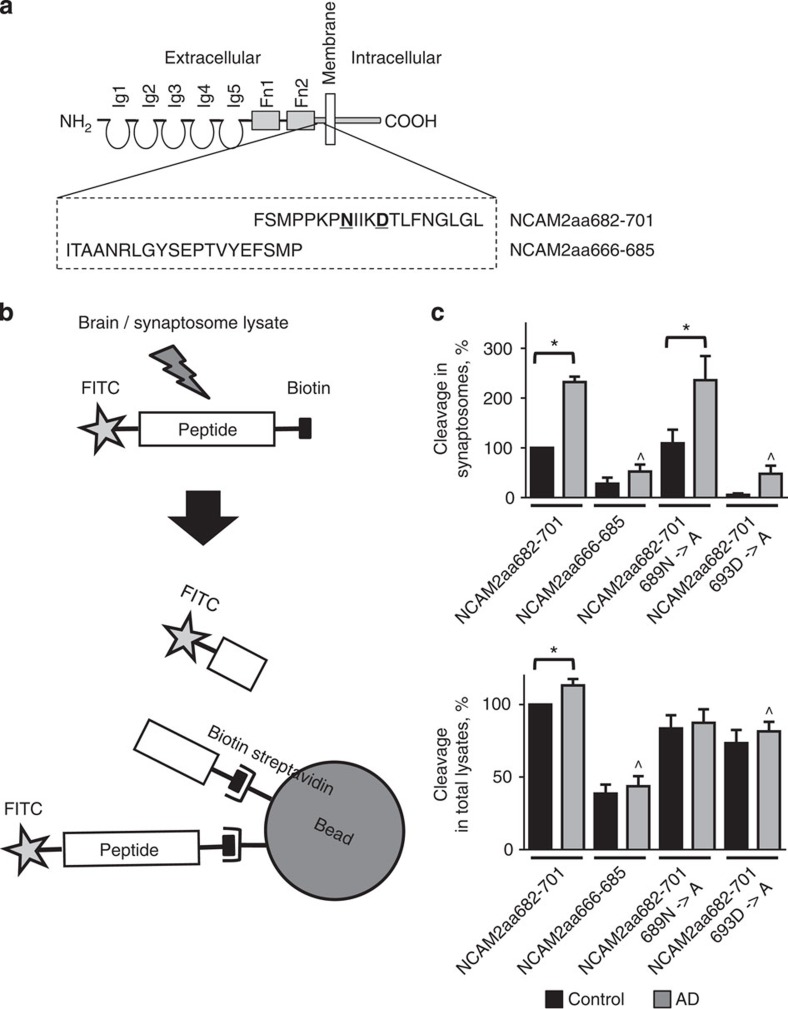
Cleavage of the membrane-adjacent extracellular fragment of NCAM2 is increased in AD brains. (**a**) Diagram showing the structure of NCAM2. Ig, immunoglobulin-like domain; Fn, fibronectin type III domain. Peptides used in the cleavage assay and corresponding to aa682-701 (NCAM2aa682-701) and aa666-685 (NCAM2aa666-685) of human NCAM2 are shown below. Asparagine 689 and aspartic acid 693 exchanged to alanine in the cleavage assay shown in **c** are highlighted in bold. (**b**) Scheme of the peptide cleavage assay. Peptides labelled with FITC and biotin at the N- and C-terminus, respectively, were incubated either with the total brain lysate or lysate of synaptosomes. Non-cleaved peptides and biotin-containing fragments of the cleaved peptides were removed using streptavidin-coated beads. The remaining FITC fluorescence was used as an estimate of peptide cleavage. (**c**) Graphs show the efficiency of the peptide cleavage (mean+s.e.m.) with the fluorescence signals for NCAM2aa682-701 in controls set to 100%. Lysates from eight controls and eight AD patients were analysed. Note that the efficiency of NCAM2aa682-701 cleavage is higher in AD cases and particularly in synaptosomes. NCAM2aa682-701 cleavage is reduced by mutating aspartic acid 693. *P<0.05 (compared as indicated), ^*P*<0.05 (compared with NCAM2aa682-701 cleavage in AD) analysis of variance with Tukey's multiple comparisons test.

**Figure 3:**
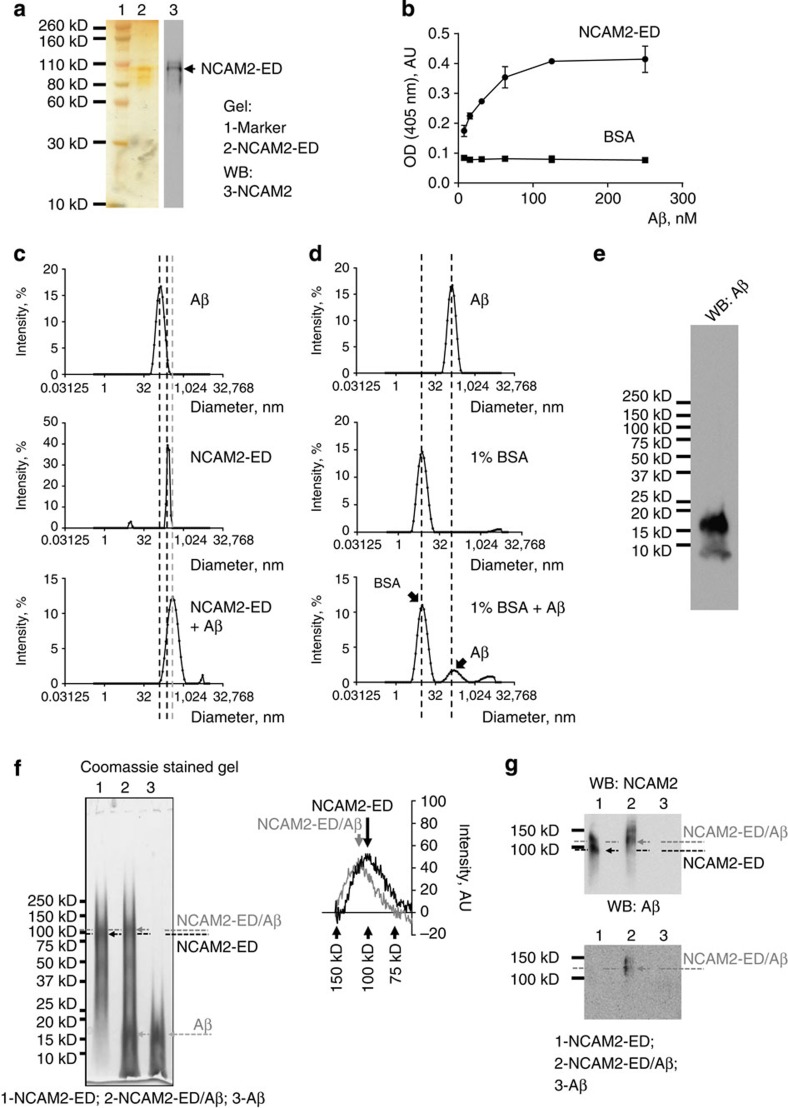
The extracellular domain of NCAM2 binds to Aβ. (**a**) Recombinant extracellular domains of NCAM2 (NCAM2-ED) analysed by silver staining and western blot with antibodies against the extracellular domain of NCAM2. (**b**) NCAM2-ED immobilized on the plastic surface of 96-well plates was assessed by ELISA for its ability to bind increasing concentrations of Aβ_1-42_. Mean+s.e.m. (*n*=3) OD values from a representative experiment are shown. Note that Aβ_1-42_ binds to NCAM2-ED but not to BSA in a concentration-dependent manner. The experiment was performed five times with the same effect. (**c**) DLS analysis of the hydrodynamic diameters of the protein particles in the solutions containing Aβ_1-42_ oligomers alone, NCAM2-ED alone or a mixture of Aβ_1-42_ oligomers and NCAM2-ED. Note that the particle size peaks (marked by black dashed lines) are shifted to a larger hydrodynamic diameter (grey dashed line) in the solution containing a mixture of Aβ_1-42_ oligomers and NCAM2-ED when compared with solutions containing Aβ_1-42_ oligomers or NCAM2-ED alone. (**d**) DLS analysis of the hydrodynamic diameters of the protein particles in the solutions containing Aβ_1-42_ oligomers alone, BSA alone or a mixture of Aβ_1-42_ oligomers and BSA. Note that the particle size peaks (marked by dashed lines) are not shifted when Aβ_1-42_ oligomers are incubated with BSA. The experiment in **c**,**d** was performed twice with the same effect and a representative experiment is shown. (**e**) Western blot analysis of the Aβ_1–42_ oligomer preparation used in this study. (**f**,**g**) PAGE (**f**) and western blot (**g**) analyses of the probes containing NCAM2-ED, NCAM2-ED incubated with Aβ_1-42_, or Aβ_1-42_ performed under non-reducing conditions. Dashed lines and arrows indicate the position of the peaks in the labelling density determined by the densitogram analysis. A fragment of the densitogram covering 75–150 kDa range is shown in **f**. Note that the density peak in the NCAM2 band in the probes incubated with Aβ_1-42_ is shifted to the higher molecular weight (grey arrows) when compared with the NCAM2 band in the absence of Aβ_1-42_ (black arrows). Full-length versions of the western blots are shown in Supplementary Fig. 9. OD, optical density. AU, arbitrary units.

NCAM2 is a membrane protein (Fig. 2a). Its proteolysis results in the release of the free, non-membrane-attached soluble fragments^22^. To investigate whether a reduction in the synaptic levels of NCAM2 is accompanied by changes in NCAM2 proteolysis, soluble protein fractions were isolated from the cerebellum, superior temporal cortex and hippocampus of control and AD brains. Western blot analysis of these fractions showed enrichment of the soluble marker-protein GAPDH as well as NCAM2-positive bands (Fig. 1c), which were detected at a lower molecular weight compared with NCAM2-positive bands in synaptosomes (Fig. 1c). Since the antibodies used in our analysis were against the extracellular domain of NCAM2 (NCAM2-ED), the ∼100-kDa NCAM2 fragment detectable in the soluble protein fractions likely represented NCAM2-ED. In agreement, the molecular weight of recombinant NCAM2-ED was also ∼100 kDa (Fig. 3a).

The levels of the soluble NCAM2 fragments relative to the NCAM2 levels in synaptosomes were significantly increased in AD hippocampus (Fig. 1c,d). Similar results were obtained when the levels of NCAM2 in the soluble protein fraction were normalized to the levels of GAPDH (Fig. 1e). The levels of the soluble NCAM2 were not statistically significantly different in the temporal cortex and cerebellum in AD brains (Fig. 1c–e). These results suggested increased proteolysis of NCAM2 and release of NCAM2-ED from the synaptic plasma membrane in the hippocampus in AD brains.

### Cleavage of NCAM2aa682-701 is increased in AD brains

To confirm that amino acid sequences within NCAM2, which are proximal to the extracellular leaflet of the membrane, can be cleaved in the brain tissue, peptides corresponding to the amino acid sequences within this region of NCAM2 (Fig. 2a) were analysed in a cleavage assay (Fig. 2b). In this assay, peptides labelled with FITC and biotin at the N- and C-terminus, respectively, were incubated with lysates of homogenates or synaptosomes from control or AD hippocampus. Non-cleaved peptides were removed using streptavidin beads. Levels of FITC groups released by the peptide cleavage were analysed by measuring FITC fluorescence (Fig. 2b). This analysis showed that the release of FITC groups from the most membrane proximal NCAM2aa682-701 fragment was significantly higher than from the adjacent NCAM2aa666-685 fragment (Fig. 2c), indicating that this sequence is susceptible to cleavage. Cleavage efficiency of NCAM2aa682-701 was markedly increased in AD samples with the strongest effect observed in the lysates of synaptosomes when compared with the total homogenate lysate (Fig. 2c). Cleavage of NCAM2aa682-701 was reduced when aspartic acid 693 was changed to alanine, but not after mutating asparagine 689 to alanine (Fig. 2c). Thus, we found that the extracellular region of NCAM2 is cleaved at amino acids 685–701, with aspartic acid at position 693 being essential. Furthermore, the cleavage rate at this site is increased in synapses of AD hippocampus.

### NCAM2 binds to Aβ *in vitro*

In search for possible mechanisms of the increased proteolysis of synaptic NCAM2 in AD, we determined whether purified recombinant NCAM2-ED (Fig. 3a) binds to Aβ_1-42_ *in vitro* using ELISA. Aβ_1-42_ bound to NCAM2-ED immobilized on plastic in a concentration-dependent manner (Fig. 3b). No binding to bovine serum albumin (BSA) used as a negative control was observed. Hence, NCAM2 can directly associate with Aβ_1-42_.

To further understand the nature of the complexes formed by NCAM2-ED and Aβ_1-42_, the sizes of the protein particles formed by NCAM2-ED or Aβ_1-42_ alone or when NCAM2-ED and Aβ_1-42_ were incubated together were measured by using dynamic light scattering. This analysis showed that Aβ_1-42_ formed particles with the hydrodynamic diameter of ∼140 nm (Fig. 3c), as previously reported for Aβ oligomers^23^. In agreement, SDS–polyacrylamide gel electrophoresis (PAGE) and western blot analysis of the Aβ_1-42_ preparation with human Aβ-specific antibodies (6E10, Covance) showed a band at ∼18 kDa corresponding to Aβ_1-42_ tetramers and a minor band at ∼4.5 kDa corresponding to Aβ_1-42_ monomers but no higher molecular weight bands (>40 kDa) corresponding to protofibrils (Fig. 3e)^24^. The hydrodynamic diameter of the particles formed by NCAM2-ED was ∼260 nm (Fig. 3c). NCAM2-ED incubated with Aβ_1-42_ oligomers formed larger protein particles with the hydrodynamic diameter of ∼440 nm (Fig. 3c), indicating binding of NCAM2 to Aβ_1-42_. In contrast, incubation of Aβ_1-42_ oligomers with BSA did not induce any shift in the size indicating no particles were formed by BSA and Aβ_1-42_ (Fig. 3d).

To confirm the dynamic light scattering data, NCAM2-ED incubated with or without Aβ_1-42_ oligomers was analysed by PAGE under non-reducing conditions. Analysis of the densitograms of the Coomassie-stained gels showed that incubation with Aβ_1-42_ resulted in a slight ∼15–20 kDa shift of the NCAM2-ED band to the upper molecular weight with no higher molecular complexes observed (*n*=3 experiments; Fig. 3f). A similar shift was observed when the probes were analysed by western blot with antibodies against NCAM2 (Fig. 3g). Furthermore, western blot analysis with antibodies against Aβ confirmed that the shifted band was also positive for Aβ (Fig. 3g). Since the shift corresponded to the molecular weight of the tetrameric Aβ_1-42_ oligomers (Fig. 3e), our data indicate that Aβ_1-42_ oligomers bind to NCAM2.

**Figure 4:**
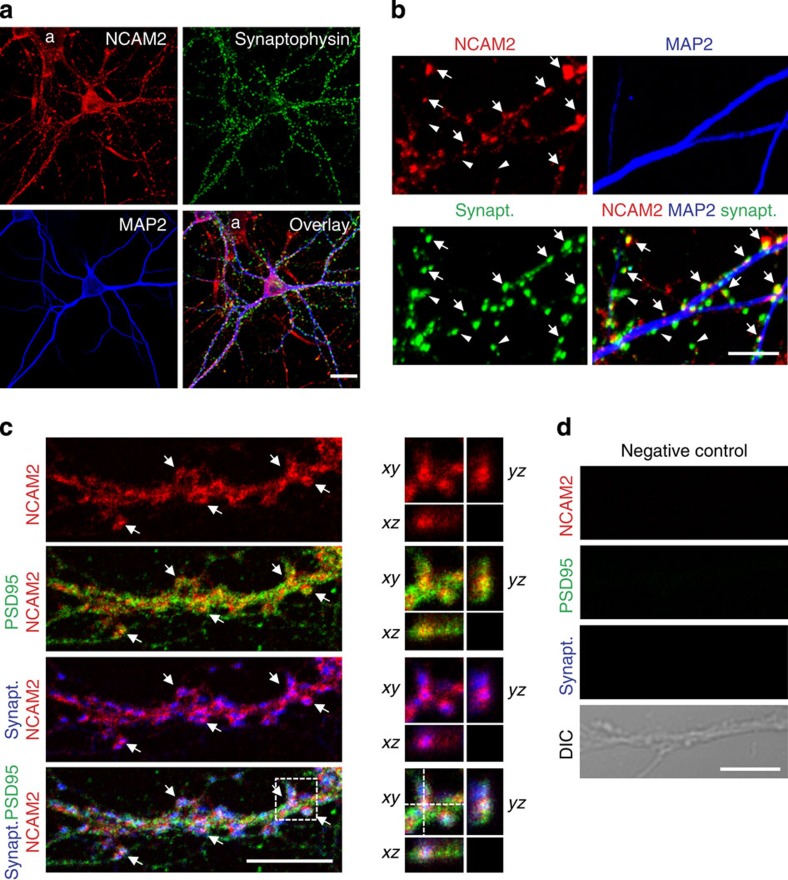
NCAM2 accumulates in excitatory synapses of cultured hippocampal neurons. (**a**) Low-magnification image of a cultured hippocampal neuron labelled by indirect immunofluorescence with antibodies against NCAM2, synaptophysin and MAP2. Note expression of NCAM2 along MAP2 positive dendrites. NCAM2 is also expressed in astrocytes (marked **a**) which are present in these cultures. Scale bar, 20 μm. (**b**) High-magnification image of dendrites of neurons co-labelled with antibodies against NCAM2, synaptophysin and MAP2. Arrows show clusters of NCAM2 partially overlapping with synaptophysin accumulations. NCAM2-negative synapses are also observed (arrowheads). Scale bar, 10 μm. (**c**) High-magnification image of a dendrite of a cultured hippocampal neuron labelled with antibodies against NCAM2, synaptophysin and PSD95. NCAM2 clusters partially overlap with accumulations of PSD95 and synaptophysin (arrows). Scale bar, 10 μm. Three-dimensional analysis of the co-localization within the outlined area is on the right. *Z*-stack has been acquired with 0.15 μm steps. The *xz* and *yz* sections along the dashed lines on the *xy* image are shown. Note co-localization of the NCAM2 cluster with synaptic markers. (**d**) Negative control, that is, labelling performed without primary antibodies, is shown. Scale bar, 10 μm.

To analyse whether NCAM2 can also associate with Aβ_1-42_ oligomers at the cell surface of neurons, we used cultured mouse hippocampal neurons. First, we confirmed synaptic localization of NCAM2 in these neurons by using immunofluorescence labelling. Labelling with antibodies against NCAM2 showed that it was distributed along dendrites that stained for the dendritic marker-protein MAP2 (Fig. 4a). Higher magnification visualized clusters of NCAM2 co-localizing with the presynaptic marker synaptophysin (Fig. 4b). Interestingly, NCAM2 was enriched in a subset of synapses (Fig. 4b). Co-labelling with antibodies against the postsynaptic marker of excitatory synapses, PSD95, showed that NCAM2 co-localized with PSD95-positive synapses (Fig. 4c, Pearson's correlation coefficient for NCAM2 and PSD95 labelling was 0.752±0.008, *n*=23 neurons analysed). These observations indicate that similarly to its distribution in human brain, NCAM2 is enriched in synapses of mouse hippocampal neurons.

**Figure 5:**
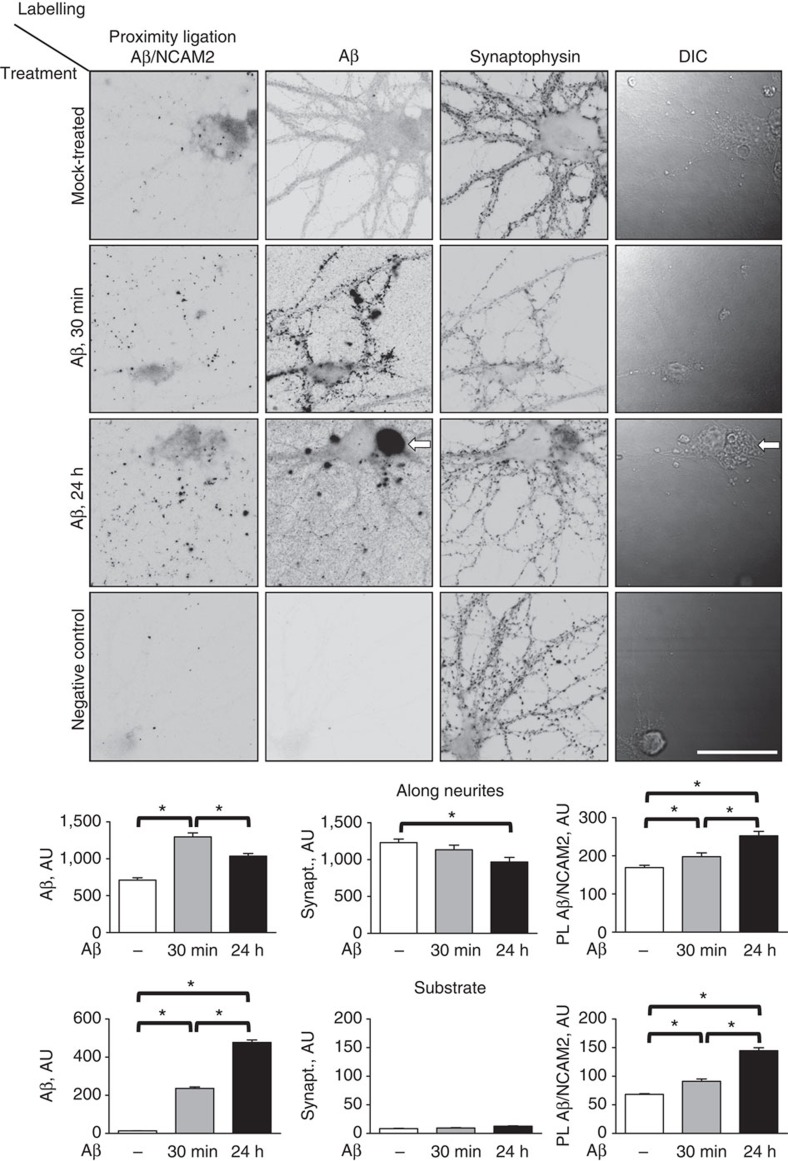
Aβ_1–42_ oligomers bind to NCAM2 at the cell surface of neurons. Representative images of cultured hippocampal neurons, which were either mock-treated or incubated with Aβ_1-42_ oligomers for 30 min or 24 h. Aβ_1-42_/NCAM2 complexes at the cell surface were detected by PL using antibodies against Aβ_1-42_ and the extracellular domain of NCAM2 applied to detergent non-permeabilized neurons. Neurons were then co-labelled with antibodies against Aβ_1-42_ and synaptophysin used to visualize neurons. Differential interference contrast images (DIC) and inverted grey scale fluorescence images are shown. Note endogenous APP labelling in mock-treated neurons and increased levels of Aβ_1-42_ immunoreactivity and NCAM2/Aβ_1-42_ proximity ligation reaction products along neurites of neurons treated with Aβ_1-42_ oligomers for 30 min. NCAM2/Aβ_1-42_ proximity ligation reaction products and Aβ_1-42_ oligomers were observed along neurites and also adsorbed to the substrate around neurons treated with Aβ_1-42_ oligomers for 24 h. A dead neuron with disrupted morphology of the soma, fragmented dendrites and highly positive for Aβ_1-42_ is marked with an arrow. Graphs show levels of Aβ_1-42_ and synaptophysin immunoreactivity and Aβ_1-42_/NCAM2 proximity ligation products measured along neurites and randomly sampled areas around neurons (mean+s.e.m., *n*>50 neurons analysed in each group). *P*<0.05, analysis of variance with uncorrected Fisher's least square difference test. The experiment was performed twice with the same effect. Scale bar, 40 μm.

Next, neurons were treated with 0.5 μM Aβ_1-42_ oligomers or equivalent volume of the neuronal culture medium used to dilute oligomers for 30 min or 24 h. Neurons were then subjected to a proximity ligation (PL) assay using antibodies against Aβ_1-42_ (sc-28365; Santa Cruz Biotechnology) and antibodies against the extracellular domain of NCAM2 (ref. 19) to detect complexes of NCAM2 and Aβ_1-42_. Neurons were co-labelled for synaptophysin and Aβ_1-42_ to visualize synapses and neuronal morphology. Proximity ligation produced a weak reaction in mock-treated control neurons (Fig. 5), suggesting that NCAM2 associates with endogenous APP present in cultured hippocampal neurons (see also Fig. 8f). Neurons incubated with Aβ_1-42_ oligomers for 30 min presented with a strong punctuated Aβ immunoreactivity along dendrites partially overlapping with synaptophysin accumulations (Fig. 5), indicating that Aβ_1-42_ oligomers bound to the cell surface at synapses, as described previously^13^. This was accompanied by a significant increase in the NCAM2/Aβ_1-42_ PL signal (Fig. 5). Interestingly, the NCAM2/Aβ_1-42_ PL products were observed not only along neurites but also on the substrate in neurite-free areas (Fig. 5). In neurons incubated with Aβ_1-42_ oligomers for 24 h, levels of NCAM2/Aβ_1-42_ ligation products adsorbed to the substrate around neurons were increased as compared to neurons incubated with Aβ_1-42_ for 30 min (Fig. 5). This increase in the substrate-bound ligation products was accompanied by a reduction in levels of Aβ_1-42_ immunoreactivity along neurites in neurons incubated with Aβ_1-42_ oligomers for 24 h as compared with neurons incubated with Aβ_1-42_ oligomers for 30 min (Fig. 5), suggesting that prolonged incubation with Aβ_1-42_ oligomers resulted in the dissociation of NCAM2/Aβ_1-42_ complexes from the cell surface and subsequent adsorption to the substrate. Dead neurons containing high levels of Aβ immunoreactivity, probably representing intracellular accumulations of Aβ, were also observed in cultures treated with Aβ_1-42_ oligomers for 24 h (Fig. 5), in accordance with previous reports showing that intraneuronal accumulation of Aβ_1-42_ precedes neuronal death^25^. Omission of one of the antibodies abolished the PL signal indicating the specificity of the reaction (Fig. 5). Taken together, our data suggest that Aβ_1–42_ oligomers form complexes with NCAM2 that progressively dissociate from synaptic sites into the medium.[Fig f6]

### Aβ removes NCAM2 from synapses of hippocampal neurons

**Figure 6 f6:**
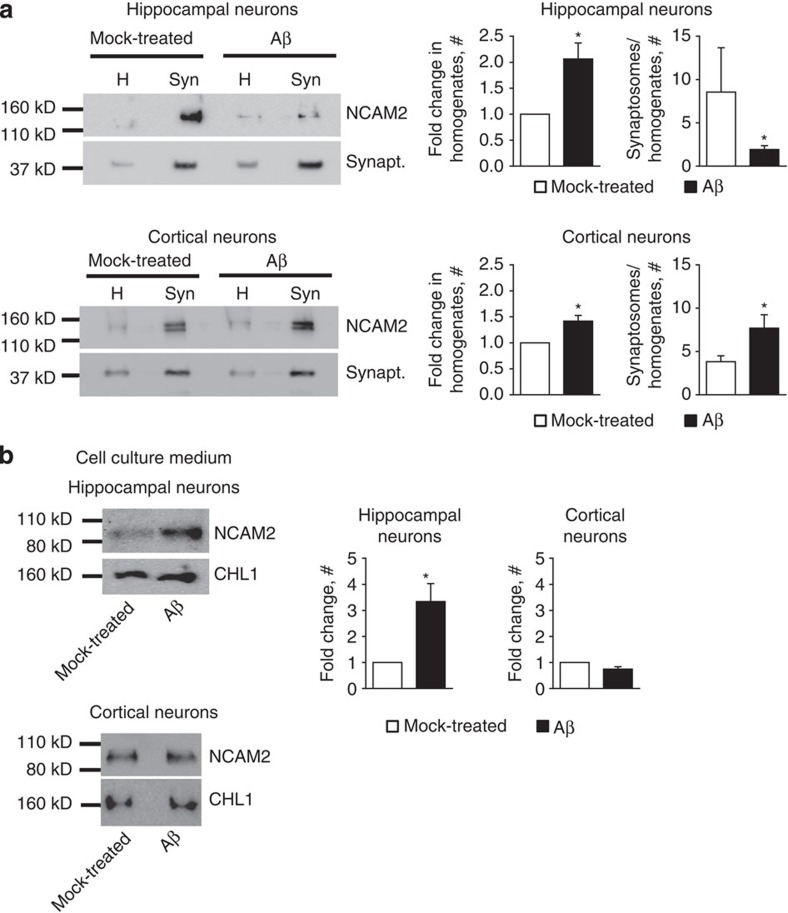
Levels of NCAM2 are reduced in synaptosomes of cultured hippocampal neurons treated with Aβ_1-42_ oligomers. (**a**) Western blot analysis of homogenates and synaptosomes isolated from cultured hippocampal or cortical neurons either mock treated or incubated with Aβ_1-42_ oligomers for 24 h. Blots were probed with antibodies against NCAM2 and synaptophysin used as a loading control. Note that NCAM2 is enriched in synaptosomes versus homogenates in hippocampal neurons and this enrichment is reduced in hippocampal neurons treated with Aβ_1-42_. Levels of NCAM2 in synaptosomes from cortical neurons are not reduced in response to Aβ_1-42_. NCAM2 levels are increased in homogenates of neurons treated with Aβ_1-42_ versus mock-treated neurons. Graphs show mean+s.e.m. fold change in NCAM2 levels in cell culture homogenates with levels in mock-treated neurons set to 1 (left panels), and mean+s.e.m. of the synaptic enrichment of NCAM2 defined as a ratio of NCAM2 levels in synaptosomes to homogenates (right panels) from *n*=5 independent experiments. *P<0.05 paired *t*-test. (**b**) Western blot analysis of the cell culture media collected from the hippocampal and cortical neurons either mock-treated or incubated with Aβ_1-42_ oligomers for 24 h. Blots were probed with antibodies against the extracellular domain of NCAM2. Note that levels of the soluble ∼100 kDa extracellular fragments of NCAM2 are increased in the culture media from cultured hippocampal neurons treated with Aβ_1-42_. Graphs show quantification of the blots (mean+s.e.m., *n*=3 independent experiments for hippocampal neurons, *n*=6 independent experiments for cortical neurons) with levels in mock-treated cultures set to 1. *P<0.05 paired *t*-test. For **a**,**b**, full-length versions of the blots are shown in Supplementary Fig. 10.

To confirm that exposure to Aβ_1-42_ oligomers affects levels of NCAM2 at synapses, we analysed levels of NCAM2 in synaptosomes isolated from control mock-treated cultured hippocampal neurons and neurons treated with Aβ_1-42_ oligomers for 24 h. Western blot analysis of synaptosomes showed that NCAM2 was highly enriched in synaptosomes isolated from control neurons (Fig. 6a), in agreement with immunocytochemical analysis (Fig. 4) and observations with human brain tissue (Fig. 1a). The synaptic enrichment of NCAM2 was significantly reduced in synaptosomes from neurons treated with Aβ_1-42_ oligomers (Fig. 6a). Levels of synaptophysin were similar in synaptosomes isolated from Aβ_1-42_- and mock-treated neurons, indicating similar isolation efficiency (Fig. 6a).

The fact that levels of NCAM2 were not significantly affected in the temporal cortex of AD-affected individuals (Fig. 1a) prompted us to analyse the effect of Aβ_1-42_ oligomers on NCAM2 levels in synapses of cortical neurons. Synaptosomes isolated from cultured cortical neurons showed NCAM2 enrichment in synapses, albeit at lower levels as compared with hippocampal neurons (Fig. 6a). Interestingly, but in line with our findings in human temporal cortex (Fig. 1a), levels of NCAM2 in synaptosomes were not reduced by exposure of cortical neurons to Aβ_1-42_ for 24 h (Fig. 6a). Western blot analysis of the cell culture lysates showed that in hippocampal and cortical neurons incubated with Aβ_1-42_ oligomers the overall levels of NCAM2 were increased when compared with NCAM2 levels in mock-treated neurons (Fig. 6a), indicating a similar compensatory reaction in both types of neurons. It is therefore possible that cortical neurons are more efficient in compensating for the Aβ-dependent loss of NCAM2 because levels of NCAM2 in synapses of these neurons are lower.

To confirm that Aβ_1-42_ induces removal of NCAM2 from the cell surface of cultured hippocampal neurons, we compared levels of the soluble proteolytic products of NCAM2 in the cell culture medium collected from control mock-treated neurons and neurons treated with Aβ_1-42_ oligomers for 24 h. Western blot analysis showed that levels of soluble NCAM2 with the molecular weight of ∼100 kDa were significantly increased in culture medium from Aβ_1-42_-treated hippocampal neurons (Fig. 6b), further indicating that Aβ_1-42_ induces removal of NCAM2 off the neuronal cell surface. In contrast, levels of the soluble proteolytic products of CHL1, another synaptic cell adhesion molecule of the immunoglobulin superfamily^26^,^27^, were not changed in the culture medium from Aβ_1-42_-treated hippocampal neurons (Fig. 6b). Incubation with Aβ_1-42_ did not increase levels of soluble NCAM2 in the culture medium from cortical neurons (Fig. 6b), suggesting that cortical neurons are more resistant to Aβ_1-42_-dependent NCAM2 proteolysis.

### Aβ binds to and removes NCAM2 from synapses in APP23 mice

**Figure 7:**
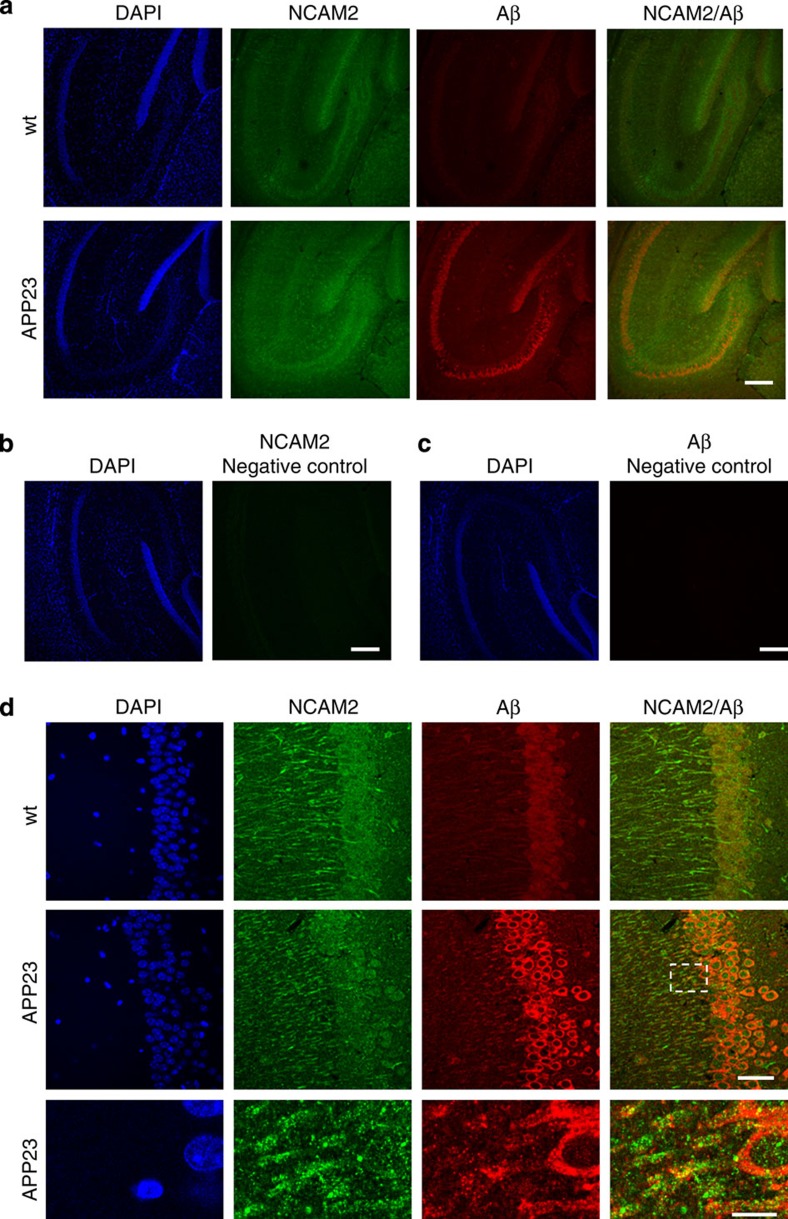
NCAM2 co-localizes with Aβ_1-42_ in brains of APP23 transgenic mice. Low-magnification confocal images of the hippocampus (**a**) and high-magnification images of the CA1 region of the hippocampus (**d**) of 9-month-old wild-type (wt) and APP23 mice (APP23) are shown. Brain sections were immunolabelled with antibodies to NCAM2 and Aβ and counter stained with DAPI. Lower panel in **d** shows a blow-up of the area outlined with dashed lines. Note accumulations of Aβ co-localizing with clusters of NCAM2 along dendrites of neurons. Negative controls (labelling performed without primary antibodies) are shown in **b**,**c**. Scale bar=200 μm (**a**–**c**), 40 μm (**d**), 10 μm (**d**, blow-up image).

Next, we used Aβ-forming human mutant APP-expressing APP23 mice to analyse whether Aβ interacts with NCAM2 in the brain *in vivo*. Immunolabelling of brain slices with antibodies to NCAM2 showed widespread NCAM2 expression in hippocampi of both wild-type and APP23 mice of different age (Fig. 7a, Supplementary Figs 3 and 4). Antibodies against human Aβ (6E10, Covance) stained neurons in the hippocampus of APP23 mice (Fig. 7a, Supplementary Fig. 3). NCAM2 and Aβ partially co-localized along dendrites of the neurons in 9-month-old APP23 mice (Fig. 7d), which did not show overt plaques (Fig. 7a), in small Aβ aggregates probably representing nascent plaques in 12-month-old APP23 mice (Supplementary Figs 3 and 4), and in small Aβ aggregates and around mature plaques in 24-month-old APP23 mice (Supplementary Figs 3 and 4). No labelling was observed when primary antibodies were omitted (Fig. 7b,c).[Fig f8]

**Figure 8 f8:**
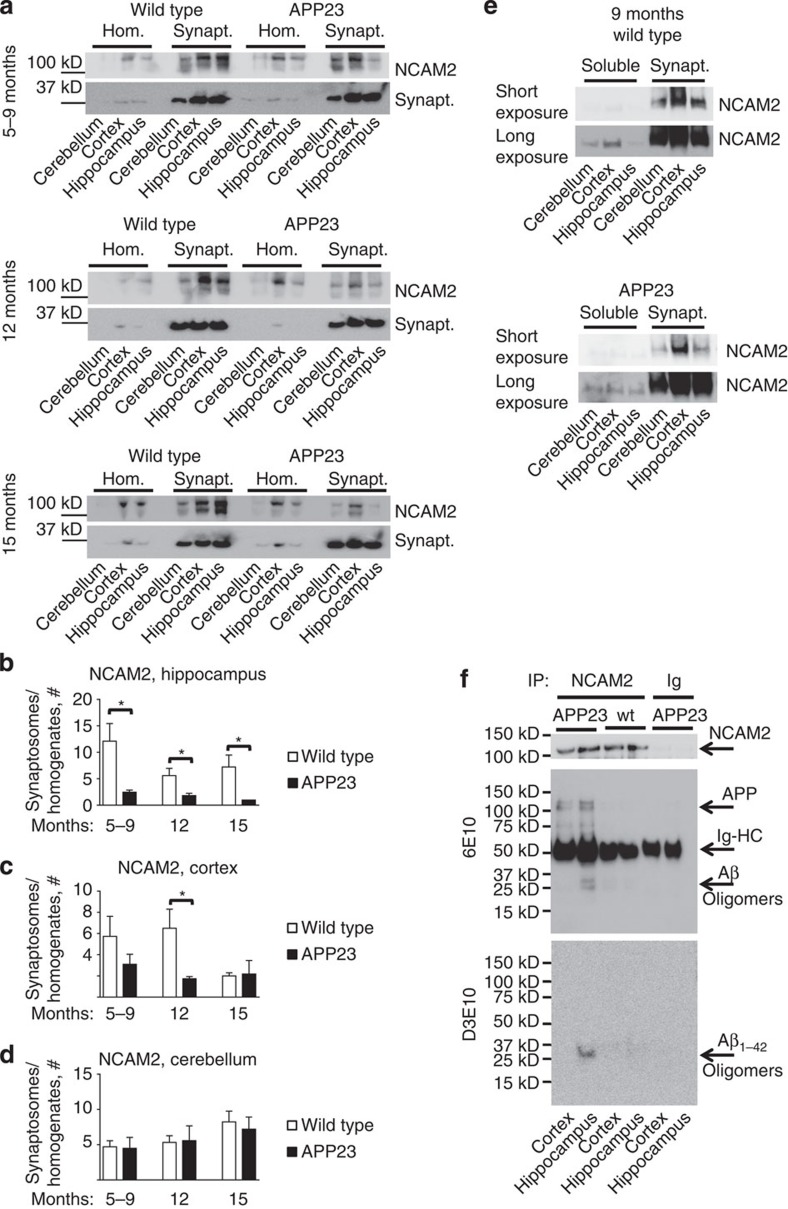
NCAM2 binds to Aβ and its synaptic accumulation is reduced in the hippocampus of APP23 transgenic mice. (**a**) Western blot analysis of the homogenates (hom.) and synaptosomes (synapt.) prepared using hippocampus, cortex and cerebellum from wild-type and APP23 transgenic (APPtg) mice. Probes were analysed with antibodies against NCAM2 and synaptophysin used as a loading control. Note reduced synaptic enrichment of NCAM2 in the hippocampus of APP23 transgenic mice. Full-length versions of the western blots are shown in Supplementary Fig. 11. (**b**–**d**) Graphs show synaptic enrichment of NCAM2 defined as a ratio of NCAM2 levels in synaptosomes and homogenates (mean+s.e.m., samples from four 5–9-month-old, four 12-month-old and two 15-month-old pairs of littermates were analysed twice by western blot). *P<0.05 paired *t*-test. (**e**) Western blot analysis of the soluble protein fraction and synaptosomes prepared using hippocampus, cortex and cerebellum from wild-type and APP23 transgenic mice. Probes were analysed with antibodies against NCAM2. Note reduced synaptic enrichment of NCAM2 in the hippocampus of APP23 mice (short exposure) and higher levels of soluble NCAM2 fragments in the hippocampus of APP23 mice when compared with wild-type hippocampus revealed after prolonged exposure of the blot (long exposure). Full-length versions of the western blots are shown in Supplementary Fig. 12. (**f**) Western blot analysis of NCAM2 immunoprecipitates (IP) from the cortex and hippocampus of wild-type and APP23 mice. Mock IP with non-immune immunoglobulins (Ig) served as control. Membranes were analysed with antibodies to NCAM2, human APP and Aβ (6E10, Covance), or Aβ_1-42_ (D3E10, Cell Signaling). Note full-length APP and Aβ immunoreactivity in NCAM2 immunoprecipitates from APP23 mice. A band representing the heavy chain of the immunoglobulins (Ig-HC) used for immunoprecipitation and recognized by the secondary antibodies is also observed. Labelling with antibodies to NCAM2 shows NCAM2 immunoprecipitation efficiency. Full-length versions of the western blots are shown in Supplementary Fig. 13.

To analyse whether increased levels of Aβ influence synaptic accumulation of NCAM2 *in vivo*, we compared synaptic enrichment of NCAM2 in brains of APP23 mice and wild-type littermates. Western blot analysis showed that NCAM2 was highly enriched in synaptosomes from the hippocampus of wild-type mice in all ages tested (Fig. 8a). Enrichment of NCAM2 in hippocampal synaptosomes was significantly reduced in APP23 mice at all ages analysed (Fig. 8a,b). NCAM2 was also highly enriched in synaptosomes isolated from the cortex of 5–9- and 12-month-old wild-type mice (Fig. 8a). This enrichment of NCAM2 progressively declined in APP23 mice with the difference reaching statistical significance at 12 months of age (Fig. 8a,c). Interestingly, synaptic levels of NCAM2 in the cortex of 15-month-old wild-type mice declined to the level of APP23 mice, possibly reflecting ageing. Synaptic enrichment of NCAM2 in the cerebellum was not different between genotypes in mice of all ages tested (Fig. 8a,d), consistent with absence of transgene expression in the cerebellum of APP23 mice^28^. A decline in synaptic levels of NCAM2 was accompanied by an increase in soluble NCAM2 fragments in the hippocampus of APP23 mice (Fig. 8e; ratio of soluble NCAM2 to full-length synaptic NCAM2 levels in synaptosomes was 0.11±0.02 in the hippocampus of wild-type animals versus 0.24±0.05* in the hippocampus of APP23 mice, *n*=5, *P<0.05, paired *t*-test), indicating increased proteolysis of NCAM2.

Although co-localization data (Fig. 7) suggests that NCAM2 and Aβ interact, it does not exclude that NCAM2 co-localize with full-length APP or its fragments containing epitopes present in Aβ and recognized by 6E10 antibodies. To confirm that NCAM2 interacts with Aβ, co-immunoprecipitation experiments were performed. Immunoprecipitation of NCAM2 from APP23 brain extracts co-immunoprecipitated Aβ species of ∼20–37 kDa detectable with antibodies against human Aβ (6E10, Covance) (Fig. 8f). Co-immunoprecipitation efficiency was stronger in the hippocampus when compared with cortex (Fig. 8f). NCAM2 immunoprecipitates from the cortex and hippocampus of APP23 mice also contained full-length APP and ∼75 kDa products possibly representing APP proteolytic fragments containing epitopes present in Aβ and detectable with 6E10 antibody (Fig. 8f). To confirm that Aβ_1-42_ associates with NCAM2, NCAM2 immunoprecipitates were also analysed with the rabbit monoclonal antibody (D3E10, Cell Signaling) recognizing Aβ_1-42_, but not full-length APP or other Aβ species. Labelling with this antibody also showed immunoreactivity at ∼20–37 kDa in NCAM2 immunoprecipitates from APP23 hippocampus (Fig. 8f). Taken together, our results suggest that Aβ binds to NCAM2 particularly in the hippocampus and may induce removal of NCAM2 from synapses *in vivo*.

### Disruption of NCAM2 adhesion promotes synapse disassembly

**Figure 9:**
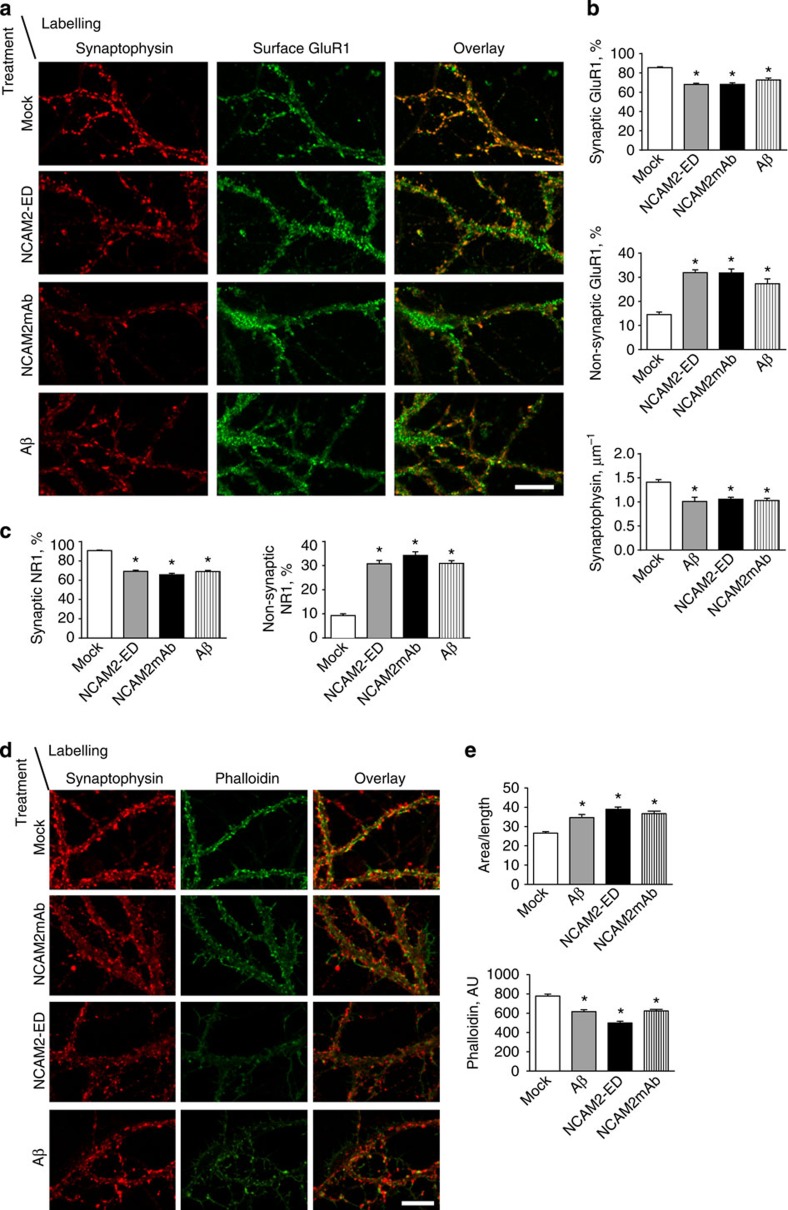
Disruption of NCAM2 functions at the neuronal cell surface promotes glutamatergic synapse disassembly. (**a**–**e**) Cultured hippocampal neurons were either mock-treated or incubated with the recombinant soluble extracellular domains of NCAM2 (NCAM2-ED), antibodies against the extracellular domain of NCAM2 (NCAM2mAb), or Aβ_1-42_ oligomers. In **a**,**b**, neurons were labelled with antibodies against the extracellular domain of GluR1 before permeabilization of membranes with detergent, and co-labelled with antibodies against synaptophysin after permeabilization of membranes with detergent. Representative images of dendrites are shown (**a**). Note co-localization of cell surface GluR1 accumulations with synaptophysin clusters in mock-treated neurons, and increased levels of non-synaptic cell surface GluR1 accumulations in neurons treated with NCAM2-ED, NCAM2mAb or Aβ_1-42_. Graphs (**b**) show the percentage of synaptic and non-synaptic GluR1 clusters relative to total number of GluR1 clusters along dendrites and numbers of synaptophysin accumulations per dendrite length (mean+s.e.m.). *P<0.0001 (analysis of variance with Dunnett's multiple comparison test, *n*>80 dendrites from 20 neurons were analysed in each group). In **c**, neurons were labelled with antibodies against the extracellular domain of NR1 before permeabilization of membranes with detergent, and co-labelled with antibodies against synaptophysin after permeabilization of membranes with detergent. Graphs show the percentage of synaptic and non-synaptic NR1 clusters relative to total number of NR1 clusters along dendrites (mean+s.e.m.). *P<0.0001 (analysis of variance with Dunnett's multiple comparison test, *n*>85 dendrites from 20 neurons were analysed). In **d**,**e**, neurons were co-labelled with fluorescent phalloidin and synaptophysin antibodies. Representative images of dendrites are shown in **d**. Note higher labelling intensity and co-localization with synaptophysin of the phalloidin-labelled polymerized actin accumulations in control neurons versus neurons treated with Aβ_1-42_, NCAM2-ED or NCAM2mAb. Note increased numbers of filopodia and lamellipodia in neurons treated with Aβ_1-42_, NCAM2-ED or NCAM2 mAb. Graphs (**e**) show ratio of the dendrite area-to-length and phalloidin labelling intensity of dendrites of neurons. Mean values+s.e.m. are shown. *P<0.0001 (analysis of variance with Dunnett's multiple comparison test, *n*=50 dendrites from 20 neurons were analysed in each group). Scale bar, 10 μm (in **a**,**d**).

Soluble NCAM2-ED contains amino acid sequences normally involved in mediating NCAM2-NCAM2 homophilic adhesion^16^. NCAM2-ED may therefore bind to membrane-localized NCAM2 and affect its interactions and functions. Since NCAM2-ED accumulates in AD brains (Fig. 1c–e) and is generated in response to Aβ_1-42_ (Fig. 6b), we determined whether application of NCAM2-ED affects synapse integrity in cultured hippocampal neurons. Neurons were incubated with recombinant NCAM2-ED (2.5 μg ml^−1^) for 24 h or mock-treated with equivalent volume of the neuronal culture medium used to dilute NCAM2-ED. To visualize synapses, neurons were labelled with antibodies against the extracellular domain of the GluR1 subunit of AMPA receptors applied before permeabilization of membranes with detergent and co-labelled with antibodies against synaptophysin applied after permeabilizing membranes with detergent. Confocal microscopy analysis showed that in mock-treated neurons over 80% of cell surface GluR1 clusters overlapped with synaptophysin accumulations and were thus identified as synaptic (Fig. 9a,b). The number of synaptic GluR1 clusters was significantly reduced in neurons incubated with NCAM2-ED (Fig. 9a,b). This reduction was accompanied by a reduction in the numbers of synaptophysin accumulations along dendrites and by an increase in the numbers of extra-synaptic GluR1 clusters identified as clusters that did not overlap with synaptophysin accumulations (Fig. 9a,b). Since NCAM2-ED can bind not only to the membrane bound NCAM2 but possibly also to other binding partners of NCAM2 at the cell surface, we also analysed whether synapse integrity is affected by antibodies, which bind specifically to the extracellular domain of NCAM2 (ref. 19, Supplementary Fig. 2). Incubation of neurons with the antibodies against the extracellular domain of NCAM2 (2.5 μg ml^−1^) for 24 h also resulted in reduced density of GluR1-positive synapses along dendrites (Fig. 9a,b). A similar effect was observed in neurons incubated with Aβ_1-42_ oligomers (0.5 μM) for 24 h (Fig. 9a,b), in agreement with previous reports^13^. Incubation with NCAM2-ED, antibodies against the extracellular domain of NCAM2 or Aβ_1-42_ oligomers also resulted in reduced numbers of synapses positive for the NR1 subunit of the NMDA receptor and an increase in numbers of extra-synaptic NR1 clusters along dendrites of neurons (Fig. 9c). Our observations thus indicate that disruption of NCAM2 functions at the cell surface results in the disassembly of glutamatergic synapses.

Since the actin cytoskeleton plays a central role in anchoring of the receptors in the postsynaptic density, we determined whether exposure to NCAM2-ED influences actin at synapses. In mock-treated control neurons, accumulations of F-actin visualized with fluorophore-labelled phalloidin co-localized with synaptophysin clusters (Fig. 9d). Numbers of synaptic phalloidin clusters and overall phalloidin intensity along dendrites were reduced in neurons treated for 24 h with NCAM2-ED or antibodies against the extracellular domain of NCAM2 (Fig. 9d,e). This effect was accompanied by the increased appearance of non-synaptic filopodia and lamellipodia along dendrites of neurons treated with NCAM2-ED or antibodies against NCAM2 (Fig. 9d), resulting in an increased ratio of dendrite area-to-length (Fig. 9e). A similar effect was observed in neurons treated for 24 h with Aβ_1-42_ oligomers (Fig. 9d,e), as described previously^13^.

### Cleavage-resistant NCAM2 reduces Aβ-dependent synapse loss

**Figure 10:**
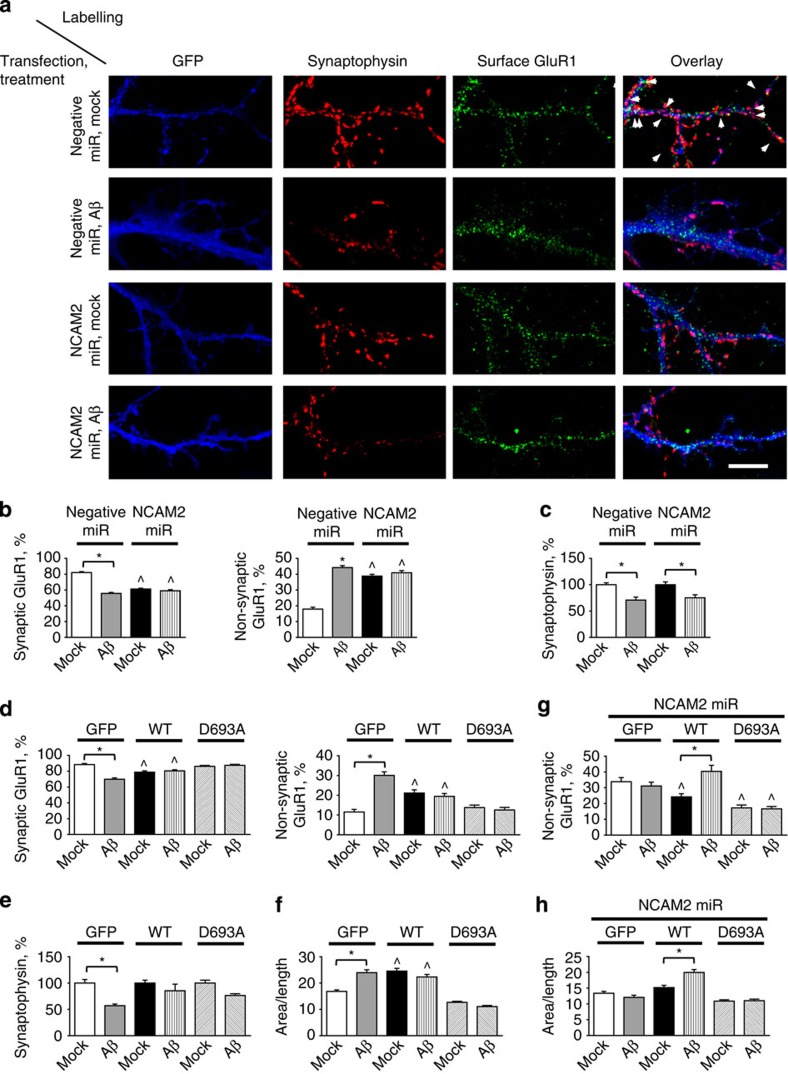
Aβ_1-42_ reduces the number of GluR1-containing synapses in the NCAM2-dependent manner. (**a**) Representative images of dendrites of cultured hippocampal neurons transfected either with control negative miRNA (negative miR) or NCAM2miR and either mock-treated or incubated with Aβ_1-42_. Transfected neurons were identified by fluorescence of GFP, which is co-expressed together with miRNA. Neurons were co-labelled with antibodies against cell surface GluR1 and synaptophysin. Note that the number of synaptic GluR1 clusters is reduced and the number of non-synaptic GluR1 clusters is increased in neurons transfected with NCAM2miR. Scale bar, 10 μm. (**b**,**c**) Graphs show mean+s.e.m. percentage of synaptic and non-synaptic GluR1 clusters relative to the total number of GluR1 clusters along dendrites (**b**) and numbers of synaptophysin accumulations per dendrite length normalized to the mean number in mock-treated neurons (**c**) for neurons described in (**a**). (**d**–**f**) Graphs show mean+s.e.m. percentage of synaptic and non-synaptic GluR1 clusters relative to the total number of GluR1 clusters along dendrites (**d**), number of synaptophysin accumulations per dendrite length normalized to the mean number in mock-treated neurons (**e**), and area/length ratio (**f**) in cultured hippocampal neurons transfected either with GFP alone or co-transfected with GFP and non-mutated NCAM2 (NCAM2WT) or NCAM2D693A mutant and either mock-treated or incubated with Aβ_1-42_. (**g**,**h**) Graphs show mean+s.e.m. percentage of non-synaptic GluR1 clusters relative to the total number of GluR1 clusters along dendrites (**g**) and area/length ratio (**h**) in cultured hippocampal neurons co-transfected with NCAM2 miR and either GFP, non-mutated NCAM2 (WT) or NCAM2D693A mutant (D693A) and either mock-treated or incubated with Aβ_1-42_. In **b**–**h**, *P<0.01 (compared as indicated), ^*P*<0.01 (compared with mock-treated neurons transfected with negative miR (**b**), GFP (**d**–**f**) or co-transfected with NCAM2miR and GFP (**g**–**h**)), analysis of variance with Tukey's multiple comparison test, *n*>50 dendrites from 20 neurons were analysed in each group.

Since Aβ_1-42_ induces removal of NCAM2 from synapses, we determined whether knockdown of NCAM2 expression by transfection of neurons with targeted miRNA (NCAM2miR) alters synapse numbers. Immunofluorescence analysis of transfected neurons showed that numbers of synaptic cell surface GluR1 clusters were reduced while numbers of extra-synaptic cell surface GluR1 clusters were increased in neurons transfected with NCAM2miR when compared with neurons transfected with control miRNA (Fig. 10a,b). When neurons transfected with control miRNA were incubated with Aβ_1-42_ oligomers for 24 h, numbers of synaptic GluR1 clusters declined and numbers of extra-synaptic GluR1 clusters increased to the levels observed in NCAM2miR transfected neurons (Fig. 10a,b). Administration of Aβ_1-42_ oligomers to NCAM2miR-transfected neurons did not increase numbers of extra-synaptic GluR1 clusters any further (Fig. 10a,b), indicating that the effects of NCAM2 knockdown and Aβ_1-42_ are not additive. Transfection with NCAM2miR did not significantly reduce the effect of Aβ_1-42_ on the overall numbers of synaptophysin-positive synaptic boutons along dendrites (Fig. 10c).

To analyse whether overexpression of NCAM2 changes the responsiveness of neurons to Aβ, neurons were transfected with DNA coding for non-mutated human NCAM2 or cleavage-resistant NCAM2 with aspartic acid 693 in the cleavage site mutated to alanine (NCAM2D693A). Transfection with both NCAM2 constructs resulted in expression of the NCAM2 proteins recognized with the NCAM2 antibodies at the expected molecular weight (Supplementary Fig. 2C). In neurons transfected with non-mutated NCAM2, the percentage of non-synaptic GluR1 clusters was increased when compared with neurons transfected with green fluorescent protein (GFP) only (Fig. 10d), possibly due to cleavage of overexpressed NCAM2 and therefore increased levels of NCAM2-ED, which affects numbers of GluR1-containing synapses (Fig. 9b). In agreement, the neurite area/length ratio was increased in NCAM2- versus GFP-transfected neurons (Fig. 10f), as observed for neurons treated with NCAM2-ED (Fig. 9d, e). Furthermore, these effects were blocked in neurons transfected with NCAM2D693A (Fig. 10d,f), indicating that cleavage of NCAM2 is required. In neurons transfected with NCAM2D693A, the Aβ_1-42_-dependent increase in numbers of extra-synaptic GluR1 clusters was blocked (Fig. 10d), and Aβ_1-42_-dependent reduction in the overall numbers of synaptic accumulations along dendrites was partially inhibited (Fig. 10e).

Because overexpression of non-mutated NCAM2 induced changes obscuring the effects of Aβ, we also analysed whether responsiveness to Aβ is restored in neurons, in which human NCAM2 was expressed on the NCAM2-negative background achieved by knockdown of expression of mouse NCAM2 using mouse NCAM2-specific NCAM2miR. Analysis of the immunofluorescence images of transfected neurons labelled with antibodies against NCAM2 showed that in neurons transfected with NCAM2miR only the levels of NCAM2 were reduced to 25.4±2.1% of NCAM2 levels in control neurons. By testing different concentrations of DNAs, we found that in neurons co-transfected with DNAs coding for NCAM2miR and non-mutated human NCAM2 or human NCAM2D693A at 1:1 ratio the levels of NCAM2 immunofluorescence were increased to 72.9±5.5% and 62.8±6.4% of the levels in control neurons, respectively. The percentage of non-synaptic GluR1 clusters was reduced in neurons co-transfected with NCAM2miR and non-mutated NCAM2 or NCAM2D693A when compared with neurons transfected with NCAM2miR only (Fig. 10g). Application of Aβ_1-42_ for 24 h induced an increase in the percentage of non-synaptic GluR1 clusters (Fig. 10g) and an increase in the neurite area/length ratio (Fig. 10h) in neurons co-transfected with non-mutated NCAM2. This effect was, however, blocked in neurons co-transfected with NCAM2D693A (Fig. 10g,h).

Taken together, our results indicate that Aβ affects the numbers of GluR1-containing glutamatergic synapses in a NCAM2-dependent manner.

## Discussion

Alzheimer's disease is characterized by loss of synapses, which is the strongest correlate of cognitive decline^11^,^29^,^30^,^31^,^32^ and possibly one of the earliest events in AD pathogenesis^30^,^33^. Synapses are long lasting contacts between neurons, which are stabilized by a number of cell adhesion molecules that concentrate in pre- and postsynaptic membranes^2^,^5^. Cell adhesion molecules play an essential role in maintaining synapse functionality and stability. Although cell adhesion molecules of many families are required for the synapse integrity^8^,^10^, elimination of even one type of synaptic cell adhesion molecule is often sufficient to induce abnormalities in synapse ultrastructure and protein composition^6^,^7^. In the present study, we show that levels of the synaptic cell adhesion molecule NCAM2 are markedly reduced in hippocampal synapses in AD brains and Aβ-forming APP23 mice. Our observations that disruption of NCAM2 interactions at the cell surface, knockdown of NCAM2 expression and Aβ exposure result in reduced numbers of glutamatergic synapses in hippocampal neurons suggest that abnormalities in NCAM2-mediated synaptic adhesion contribute to synapse loss in AD.

Although the mechanisms of synapse disassembly in AD remain poorly understood, previous studies indicated that synapse loss can be linked to Aβ-induced toxicity^12^,^34^,^35^. Our observations showing that synaptic levels of NCAM2 are similarly reduced in APP23 mice and in cultured hippocampal neurons from wild-type mice exposed to Aβ argue in favour of Aβ-dependent mechanisms in the disruption of NCAM2-mediated synaptic adhesion. We however do not exclude that other factors, such as disrupted trafficking of NCAM2 to synapses, may also contribute to the reduction of NCAM2 levels at synapses. Strikingly, the effects of Aβ on synaptic targeting of NCAM2 were particularly strong in hippocampal but not cortical or cerebellar neurons. The enhanced susceptibility of synaptic NCAM2 to Aβ-dependent proteolysis may therefore contribute to selective vulnerability of the hippocampus to AD.

Our observations that NCAM2 directly interacts with synthetic Aβ_1-42_, that Aβ_1-42_ forms a molecular complex with NCAM2 at the neuronal cell surface and that complexes of NCAM2 and oligomers of Aβ can be isolated from APP23 mouse brains, indicate that NCAM2 may function as a previously unrecognized receptor for Aβ at the neuronal cell surface. Previous studies have shown that Aβ can also bind to other cell adhesion molecules at the neuronal cell surface, among which are the prion protein^36^ and L1^37^. In addition, a number of cell adhesion molecules have been shown to interact with APP, including the neural cell adhesion molecule 1 (NCAM1)^38^ and TAG1 (ref. 39). It remains to be investigated whether the NCAM2/Aβ complex comprises other adhesion molecules and cell surface proteins. Interestingly, NCAM1, a homologue of NCAM2, binds to prion protein^40^ and L1 (ref. 41). However, in spite of homology to NCAM2, NCAM1 binds to a region of APP which is different to the Aβ-containing region^38^.

The fact that NCAM2 directly binds to Aβ suggests that this event may predispose NCAM2 to proteolysis and shedding of its extracellular domain. Aβ-derived diffusible ligands, a form of Aβ_1-42_ used in our study, have been previously shown to attach to the postsynaptic sites of excitatory synapses and induce removal of cell surface proteins^13^. How exactly Aβ induces NCAM2 proteolysis remains to be investigated, but interestingly, we could show site-specific cleavage of NCAM2 is enhanced in AD synapses. Importantly, Aβ has been shown previously to inhibit shedding of another synaptic cell adhesion molecule, N-cadherin^42^, an observation which indicates that Aβ does not induce overall degradation of the synaptic cell adhesion machinery but rather target-specific components, including NCAM2.

Notably, we found that extracellular domains of NCAM2 accumulate in brains of AD patients. In addition to enhanced proteolysis, an increase in the levels of soluble NCAM2 may also be related to overall increased expression of NCAM2 triggered by exposure to Aβ. A similar increase in levels of expression were observed for cellular prion protein, another key player in Aβ toxicity^36^, expression of which is increased in response to incubation with Aβ^43^. Our observation that incubation of cultured hippocampal neurons with recombinant extracellular domains of NCAM2 results in a reduction of the numbers of glutamatergic synapses suggests that proteolytic products of NCAM2 may exacerbate the effect of Aβ by interfering with NCAM2-mediated homophilic interactions and promoting further disassembly of synaptic contacts. In addition to the disruption of NCAM2-mediated adhesion, binding of the extracellular domains of NCAM2 may influence the intracellular cytoskeleton by activating intracellular signalling, which can be induced by other members of this family of cell adhesion molecules in response to extracellular ligand binding^44^.

Taken together, we show that Aβ induces synaptic loss and proteolysis of NCAM2 in cell culture and APP transgenic mouse models, providing a mechanistic explanation for synaptic NCAM2 changes in AD brains. The detrimental effects of proteolyically cleaved extracellular NCAM2 on synapses may augment the Aβ toxicity in the pathogenesis of AD. The exact molecular mechanisms underlying Aβ-induced NCAM2 changes, and to which degree it contributes to onset and progression of disease remains to be established. Nevertheless, our data reveal a new role of NCAM2 in AD that warrants further investigation.

## Methods

### Antibodies

Rat monoclonal antibodies against the extracellular domain of NCAM2 (MAB778; R&D Systems) were used at 2.5 μg ml^−1^ for immunocytochemistry (IC) and in experiments aimed to disrupt NCAM2 functions in live cultured hippocampal neurons. Goat polyclonal antibodies against the extracellular domain of NCAM2 (sc-51336; Santa Cruz Biotechnology) were used at 4 μg ml^−1^ for IC, immunohistochemistry (IH) and in the PL assay. Mouse monoclonal antibodies against the extracellular domain of NCAM2 (sc-136328; Santa Cruz Biotechnology) were used for IC (2 μg ml^−1^), Western blot (WB, 1 μg ml^−1^), immunoprecipitation (IP, 7 μg per 1 mg of total protein) and to immobilize NCAM2 for ELISA (10 μg ml^−1^). Goat antibodies against the extracellular domain of CHL1 (AF2147; R&D systems) were used for WB (1 μg ml^−1^); goat polyclonal and mouse monoclonal antibodies against synaptophysin (sc-7568, sc-17750; Santa Cruz Biotechnology) were used for WB (1 μg ml^−1^) and IC (4 μg ml^−1^); mouse monoclonal antibodies against actin (sc-8432; Santa Cruz Biotechnology; 1 μg ml^−1^), VGLUT1 (sc-377425; Santa Cruz Biotechnology; 1 μg ml^−1^) and VGAT (sc-393373; Santa Cruz Biotechnology; 1 μg ml^−1^) were used for WB; mouse monoclonal antibodies against PSD95 (clone K28/86, Millipore) were used for WB (0.1 μg ml^−1^) and IC (1 μg ml^−1^); mouse monoclonal antibodies against MAP2 (M4403; Sigma; 1:100) were used for IC; mouse monoclonal antibodies against Aβ (sc-28365; Santa Cruz Biotechnology) were used for WB (1 μg ml^−1^), IC (2 μg ml^−1^), PL (2 μg ml^−1^); rabbit polyclonal antibodies against Aβ (pre-diluted Aβ42 detection antibody; KHB3441 ELISA kit; Life Technologies) were used for ELISA; human-specific mouse monoclonal antibodies against Aβ (6E10) (Covance) were used for IH (1:100) and WB (1:1,000); rabbit monoclonal antibodies against Aβ_1-42_ (D3E10, Cell Signaling Technology; 1:200) were used for WB; rabbit polyclonal (A5060) and mouse monoclonal antibodies against actin (Sigma) were used for WB (1:1,000); rabbit polyclonal antibodies against the extracellular epitope of the GluR1 subunit of AMPA receptors (AGC-004; Alomone Labs, Jerusalem, Israel) were used for IC (1:100); and rabbit polyclonal antibodies against the extracellular epitope of the NR1 subunit of NMDA receptors (AGC-001; Alomone Labs) were used for IC (1:100). We also used secondary antibodies coupled to Cy2 (for IC, 1:400), Cy3 (for IC, 1:400) or Cy5 (for IC and IH, 1:400), or horseradish peroxidase (HRP) (for WB, 1:25,000) from Jackson Immunoresearch, and Alexa 555 coupled secondary antibodies (for IH, 1:1,000) from Life Technologies.

### Human brain tissue

Analysis of the human brain tissue was approved by the Human Research Ethics Committee of the University of New South Wales (permit HREC 09301). Brain tissues from ten cases per group (AD-affected individuals versus non-affected individuals with no pathology, similar age) were obtained from the Sydney Brain Bank. Cases met current diagnostic criteria for either AD or neuropathological control^45^. Case details are given in Supplementary Table 1.

### Brain tissue homogenates

Brain tissue homogenates (10%, w/v) were prepared in HOMO-A buffer: HOMO buffer (1 mM MgCl_2_, 1 mM CaCl_2_, 1 mM NaHCO_3_, 5 mM Tris, pH 7.4) containing 0.32 M sucrose, EDTA-free complete inhibitors (Roche) and 1 mM PMSF (Sigma).

### Soluble protein fractions and synaptosomes from brain tissue

Homogenates were used for synaptosome isolation as described^27^,^46^. All steps were performed at 4 °C. Briefly, homogenates were centrifuged at 1,400 g for 10 min. The supernatant and pellet were resuspended in HOMO-A buffer and centrifuged for 10 min at 700 g. The resulting supernatants were combined and centrifuged at 17,500 g for 15 min. The supernatant was centrifuged at 200,000 g for 1 h and used as the soluble fraction. The 17,500 g pellet was resuspended in HOMO-A buffer and applied on the top of a step gradient with interfaces of 0.65, 0.85, 1 and 1.2 M sucrose in HOMO buffer. The 700-g pellets were combined, adjusted to 1 M sucrose in HOMO buffer and layered on 1.2 M sucrose in HOMO buffer. HOMO-A buffer was applied on the top of the gradient. The crude synaptosomal fractions were collected at the 1 M/1.2 M interface after centrifugation for 2 h at 100,000*g* and combined. The crude synaptosomal fraction was again adjusted to 1 M sucrose and layered on the top of the 1.2 M sucrose. HOMO-A buffer was applied on the top of the gradient. After centrifugation for 2 h at 100,000*g*, synaptosomes were collected at the 1 M/1.2 M interface, resuspended in HOMO-A buffer, pulled down by centrifugation for 30 min at 100,000*g* and resuspended in HOMO-A buffer.

### Mice

Extraction of brain tissues from mice was approved by the Animal Care and Ethics Committee of the University of New South Wales (permit 12/135B). Brain tissues from one- to three-day-old wild-type C57BL/6J mice (Australian BioResources (Moss Vale, NSW, Australia)) of either sex were used for cell culture preparation. APP23 mice express human APP under control of the murine Thy1 promoter and were as described^28^. The breeding colony of APP23 mice was maintained in the Biological Resources Center at the University of New South Wales. Brain tissue from wild-type and APP23 transgenic littermates of different ages (as indicated in the text) and either sex were used for biochemical experiments.

### Cultures of hippocampal and cortical neurons

Cultures of hippocampal and cortical neurons were prepared as described^27^,^44^. Neurons were maintained in Neurobasal A medium supplemented with 2% B-27, Glutamax and 2 ng ml^−1^ bFGF-2 (all reagents from Life Technology) on glass coverslips coated with poly-D-lysine (100 μg ml^−1^, Sigma). When indicated neurons were treated for 24 h with 0.5 μM of Aβ_1–42_ peptide (AbcamBiochemicals, Cambridge, UK) in the form of Aβ-derived diffusible ligand prepared as described previously^13^,^47^,^48^, and monoclonal antibodies against the extracellular domain of NCAM2 (2.5 μg ml^−1^; R&D systems), or recombinant extracellular domains of NCAM2 (2.5 μg ml^−1^) applied in the cell culture medium.

### Immunofluorescence labelling of cultured neurons

Indirect immunofluorescence labelling was performed essentially as described previously^1^. Unless otherwise stated, all steps were performed at room temperature. Neurons on glass coverslips were fixed in 4% formaldehyde in phosphate-buffered saline (PBS) for 15 min, and then washed three times with PBS. We have shown previously that this procedure does not affect the integrity of cell surface membranes^1^. To label cell surface AMPA and NMDA receptors, neurons were then blocked in 1% BSA in PBS for 20 min. Antibodies against extracellular epitopes of GluR1 or NR1 (in 0.1% BSA in PBS) were applied to fixed but non-permeabilized cells for 1 h and detected with fluorochrome-coupled secondary antibodies applied for 45 min. The neurons were then post-fixed for 5 min in 2% formaldehyde in PBS and washed three times with PBS. To label other proteins indicated in the text, neurons were permeabilized with 0.25% Triton X-100 in PBS for 5 min, blocked with 1% BSA in PBS for 20 min, and incubated with respective primary antibodies applied in 0.1% BSA in PBS overnight at 4 °C. Neurons were then washed in PBS and incubated with corresponding fluorochrome-conjugated secondary antibodies applied for 45 min in 0.1% BSA in PBS at room temperature. When indicated, neurons were then co-labelled with Alexa Fluor 546-coupled phalloidin (Life Technologies) applied for 30 min in 0.1% BSA in PBS at room temperature. Cells were washed four times with PBS and embedded in Aqua-Poly/Mount (Polysciences, Eppelheim, Germany). Immunofluorescence images were acquired at room temperature using a confocal laser scanning microscope C1si, NIS Elements software and oil Plan Apo VC × 60 objective (numerical aperture 1.4), all from Nikon Corporation (Tokyo, Japan). Numbers of synaptic and non-synaptic clusters of AMPA and NMDA receptors were quantified in ImageJ (National Institutes of Health, USA). Clusters were automatically outlined using a threshold function of ImageJ, and clusters in which at least one pixel overlapped with a synaptophysin accumulation were counted as synaptic. Length and area of dendrites were measured in ImageJ by automatically outlining phalloidin-labelled dendrites using the threshold function of ImageJ.

### Immunofluorescence labelling of mouse brain slices

Immunofluoresecence staining was done as previously described^49^. Briefly, 9-month-old male wild-type and APP23 mice were anaesthetized and transcardially perfused with PBS followed by 4% paraformaldehyde in PBS. Brains were extracted and fixed in 4% paraformaldehyde/PBS overnight at 4 °C. Brains were then processed in a Excelsior tissue processor (Thermo), embedded in paraffin, cut into 3-μm thick sections and slide mounted. Sections were dewaxed, rehydrated to water and underwent heat-mediated antigen retrieval with citrate buffer pH6.0 using a microwave vacuum histoprocessor (Milestone). Nonspecific binding sites were blocked in a solution of PBS with 3% normal horse serum and 2% BSA, and then incubated in a cocktail of mouse monoclonal antibodies against Aβ (1:100), goat polyclonal antibodies against NCAM2 (1:50) and rabbit polyclonal antibodies against synaptophysin (1:100) at 4 °C overnight. Sections were rinsed four times in PBS and incubated in a cocktail of fluorescent secondary antibodies (donkey anti-mouse Cy5, donkey anti-goat Alexa 555, donkey anti-rabbit Cy2, 1:250) and DAPI (1:3,000) at room temperature for 1 h. Following incubation, sections were rinsed four times in PBS, coverslipped with Prolong gold anti-fade reagent (Life Technologies) and sealed with nail polish. Images were acquired using a confocal laser scanning microscope C1si, NIS Elements software and oil Plan Apo × 10 (numerical aperture 1.4) and Plan Apo VC × 60 objective (numerical aperture 1.4), all from Nikon Corporation (Tokyo, Japan).

### Proximity ligation assay

Proximity ligation experiments were performed essentially as described previously^44^,^50^. Cultured neurons were fixed in 4% formaldehyde in PBS, washed with PBS and blocked with 1% BSA in PBS for 20 min. Antibodies against the extracellular domain of NCAM2 and Aβ were applied to the cells in 0.1% BSA in PBS overnight at 4 °C. Further steps were performed using secondary antibodies conjugated with oligonucleotides (PLA probes, Olink Bioscience, Uppsala, Sweden) and Duolink II fluorescence kit (Olink Bioscience) in accordance with the manufacturer's instructions. Fluorescence images were acquired at room temperature using a confocal laser scanning microscope Nikon C1si, NIS Elements software and oil Plan Apo VC × 60 objective (numerical aperture 1.4), all from Nikon Corporation (Tokyo, Japan). Fluorescence intensities of PL products, and synaptophysin and Aβ_1-42_ labelling along neurites and in neurite-free areas were measured in ImageJ.

### Co-immunoprecipitation

Samples containing 1 mg of total protein were lysed with lysis buffer (50 mM Tris-HCl, pH 7.5, 150 mM NaCl, 1 mM Na_4_P_2_O_7_, 1 mM NaF, 2 mM Na_3_VO_4_, 1% (v/v) Triton X-100, 1 mM PMSF, EDTA-free protease inhibitor cocktail (Roche)) for 1 h at room temperature. Lysates were centrifuged for 15 min at 20,000*g* at 4 °C. Supernatants were cleared with protein A/G-agarose beads (Santa Cruz Biotechnology) for 3 h, and beads were removed by centrifugation at 600*g* for 5 min. The supernatant was incubated with the mouse monoclonal antibodies against the extracellular domain of NCAM2 or non-immune mouse IgG overnight, followed by precipitation with protein A/G-agarose beads for 3 h. The beads were pelleted and washed four times with the lysis buffer and three times with TBS. All steps were carried out at 4 °C. The proteins were finally eluted from beads with 5 × SDS sample buffer (310 mM Tris-HCl, pH 6.8, 25% (v/v) glycerol, 10% (w/v) SDS, 4.5% (v/v) β-mercaptoethanol, 0.015% (w/v) bromophenol blue) by incubating samples at 70 °C for 10 min and analysed by western blot. To eliminate detection of the bands corresponding to the light chains of the immunoglobulins used for immunoprecipitation, biotin-coupled secondary antibodies specific to Fc fragments of the primary antibodies (Sigma) were used. Signals were developed using NeutrAvidin-HRP protein conjugate (Pierce).

### Homogenates and synaptosomes from cultured neurons

Neurons maintained in culture for 20 days and treated as indicated in the text were placed on ice and washed two times with ice-cold PBS. Neurons were then scrapped in 200 μl of HOMO-A buffer and homogenized using a Potter homogenizer. Synaptosomes were prepared as described previously^26^,^51^. Briefly, the homogenate was centrifuged for 10 min at 700*g*. The supernatant was collected and centrifuged at 15,000*g* for 15 min. The pellet was then resuspended in 5 volumes of 0.1 M K_2_-tartrate (pH 7.3) and centrifuged at 5,000*g* for 5 min. The pellet collected, resuspended again in 5 volumes of 0.1 M K_2_-tartrate (pH 7.3) and centrifuged at 5,000*g* for 5 min. The pellet containing synaptosomes was resuspended in HOMO-A buffer.

### Analysis of proteolytic products of NCAM2 in culture medium

Media from neurons maintained in culture for 20 days and treated as indicated was collected and centrifuged at 200,000*g* for 1 h at 4 °C. The supernatant was then collected, diluted with the same volume of 20% trichloroacetic acid, and incubated for 30 min on ice to precipitate proteins. Precipitates were washed twice with ice-cold acetone and used for western blot analysis.

### Enzyme-linked immunosorbent assay (ELISA)

To immobilize recombinant extracellular domains of NCAM2, the plastic surface of MaxiSorp 96-well plates (Nunc, Roskilde, Denmark) was coated with antibodies against the extracellular domain of NCAM2 (10 μg per well) applied overnight at 4 °C in PBS. Wells were then washed three times with PBS containing 0.05% Tween 20, blocked with 1% BSA in PBS for 1 h at room temperature, and incubated overnight at 4 °C with recombinant extracellular domains of NCAM2 (25 nM) or BSA (25 nM) in PBS containing 0.05% Tween 20 and 0.1% BSA. Wells were washed three times with PBS containing 0.05% Tween 20 and incubated with different concentrations of Aβ_1-42_ (7.8–250 nM) diluted with 0.05% Tween 20 and 0.1% BSA. Wells were washed three times with PBS containing 0.05% Tween 20. Aβ_1-42_ bound to NCAM2 was detected with antibodies against Aβ_1-42_ followed by HRP-conjugated secondary antibodies. Protein binding was visualized by detecting HRP with the OPD reagent (Pierce, Rockford, IL, USA) that resulted in a coloured product. The amount of coloured product was quantified using an ELISA reader at 406 nm.

### Dynamic light scattering

Dynamic light scattering (DLS) measurements were performed at room temperature using Zetasizer Nano ZS (Malvern, Malvern, UK) in accordance with the manufacturer's instructions.

### Production of the extracellular domains of NCAM2

DNA coding for the extracellular domain of mouse NCAM2 (amino acids 1–698) was amplified from the mouse brain cDNA library using forward 5′-CACCATGAGCCTCCTCCTCTCCTTCTACC-3′ and reverse 5′-TTCGCTTCACAGCAATTATCTTTAATAATGTTGGGTTTT-3′ primers, cloned into pEF5/FRT/V5 directional expression vector (Life Technologies) and transfected into CHO cells (American Type Culture Collection, CHO-K1, ATCC CCL-61). Cells stably transfected with the construct were selected with Hygromycin B (Life Technology) and maintained in culture in Ham's F-12 culture medium (PAA Laboratories) with 5% foetal bovine serum (Life Technologies). Cell culture media containing the extracelluar domain of NCAM2 secreted by the cells was collected and centrifuged for 15 min at 5,000*g* at 4 °C to remove cell debris. The protein was concentrated with 40% ammonia sulphate, desalted and purified using a continuous-elution electrophoresis cell (Model 491 Prep Cell, Bio-Rad) according to the manufacturer's instructions. The purity of the protein was controlled by SDS–PAGE electrophoresis with subsequent silver staining and western blot analysis.

### Peptide cleavage assay

Hippocampal tissue or synaptosomes containing 1 mg of total protein were lysed with lysis buffer (50 mM Tris-HCl, pH 7.5, 150 mM NaCl, 1 mM Na_4_P_2_O_7_, 1 mM NaF, 1% (v/v) Triton X-100, protease inhibitor cocktail (set III, Merck)) for 30 min at room temperature. Lysates were centrifuged for 15 min at 20,000*g* at 4 °C. Peptides corresponding to amino acids 682–701 (NCAM2aa682-701) and 666-685 (NCAM2aa666-685) of human NCAM2, and mutated NCAM2aa682-701 peptides with aspartic acid 693 exchanged to alanine or asparagine 689 exchanged to alanine were purchased from Peptide 2.0 (Chantilly, VA, USA). The peptides contained FITC and biotin at the N- and C-termini, respectively. Peptides (250 ng ml^−1^ final concentration) were mixed with lysates of hippocampal tissue or synaptosomes (20 μg of total protein in 1 ml of TBS) and incubated for 1 h at room temperature. Non-cleaved peptides and biotin-containing fragments of cleaved peptide were removed by incubating lysates with hydrophilic streptavidin magnetic beads (New England BioLabs) for 1 h at room temperature. Fluorescence of FITC groups attached to fragments of cleaved peptides remaining in the solution was measured using POLARstar Omega plate reader (BMG Labtech).

### Transfection of neurons

The NCAM2 miR expression vector was developed using the BLOCK-iT PolII miR RNAi expression vector kit (Life Technologies) and designed to co-express NCAM2-specific miRNA together with emerald GFP. The following oligonucleotides were inserted into the BLOCK-iT PolII miR RNAi expression vector:

top: 5′-TGC TGT TAA GGT CAT CTC TTC TCC TCG TTT TGG CCA CTG ACT GAC GAG GAG AAG ATG ACC TTA A-3′;

bottom: 5′-CCT GTT AAG GTC ATC TTC TCC TCG TCA GTC AGT GGC CAA AAC GAG GAG AAG AGA TGA CCT TAA C-3′.

Negative control miR vector was from Life Technologies. Immunocytochemical labelling confirmed that transfection with the NCAM2 miR vector resulted in an over 80% reduction in expression of NCAM2 in cultured hippocampal neurons at 24 h after transfection (Supplementary Fig. 5).

DNA coding for the full-length transmembrane human NCAM2 and NCAM2 with aspartic acid 693 exchanged to alanine (NCAM2D693A mutant) was synthesized using the GeneArt Gene Synthesis service (Life Technologies) and subcloned into the pcDNA3 vector. Both constructs contained an HA tag inserted at the N-terminus of the protein. The expression of both constructs was confirmed by western blot and immunocytochemistry (Supplementary Fig. 2).

Hippocampal neurons were transfected before plating by electroporation using the Neon transfection system (Life Technologies).

## Additional information

**How to cite this article:** Leshchyns'ka, I. *et al.* Aβ-dependent reduction of NCAM2-mediated synaptic adhesion contributes to synapse loss in Alzheimer's disease. *Nat. Commun.* 6:8836 doi: 10.1038/ncomms9836 (2015).

## Supplementary Material

Supplementary Information Supplementary Figures 1-13 and Supplementary Table 1
