# Supplementary material for: Aβ-dependent reduction of NCAM2-mediated synaptic adhesion contributes to synapse loss in Alzheimer's disease
Source: Nat Commun. 2015 Nov 27;6:8836. doi: 10.1038/ncomms9836 (PMC4674770; doi:10.1038/ncomms9836)
Supplement: Supplementary Information — Supplementary Figures 1-13 and Supplementary Table 1 [file ncomms9836-s1.pdf]

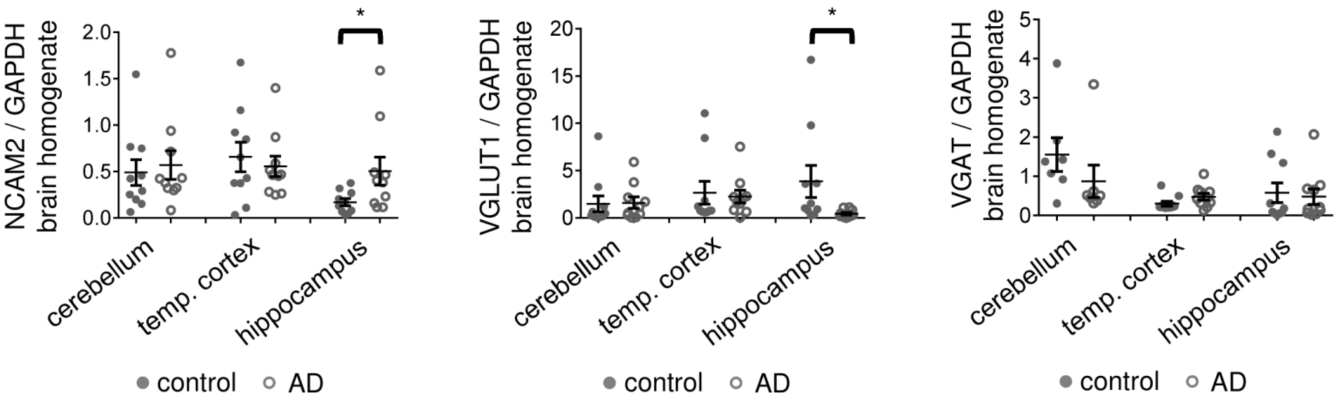

Supplementary Figure 1. Levels of NCAM2, VGLUT1, and VGAT in the hippocampus, temporal cortex and cerebellum of controls and AD-affected individuals.

Homogenates of the hippocampus, temporal cortex and cerebellum of controls and AD-affected individuals were analyzed by Western blot with antibodies against NCAM2, VGLUT1, VGAT, and GAPDH as loading control. Graphs show the protein levels for individual cases normalized to GAPDH (mean ± s.e.m., n = 10 control and n = 10 AD cases were analyzed). *$p < 0.05$, Mann Whitney test. Note that levels of NCAM2 are increased, and levels of VGLUT1 are reduced in AD hippocampus.

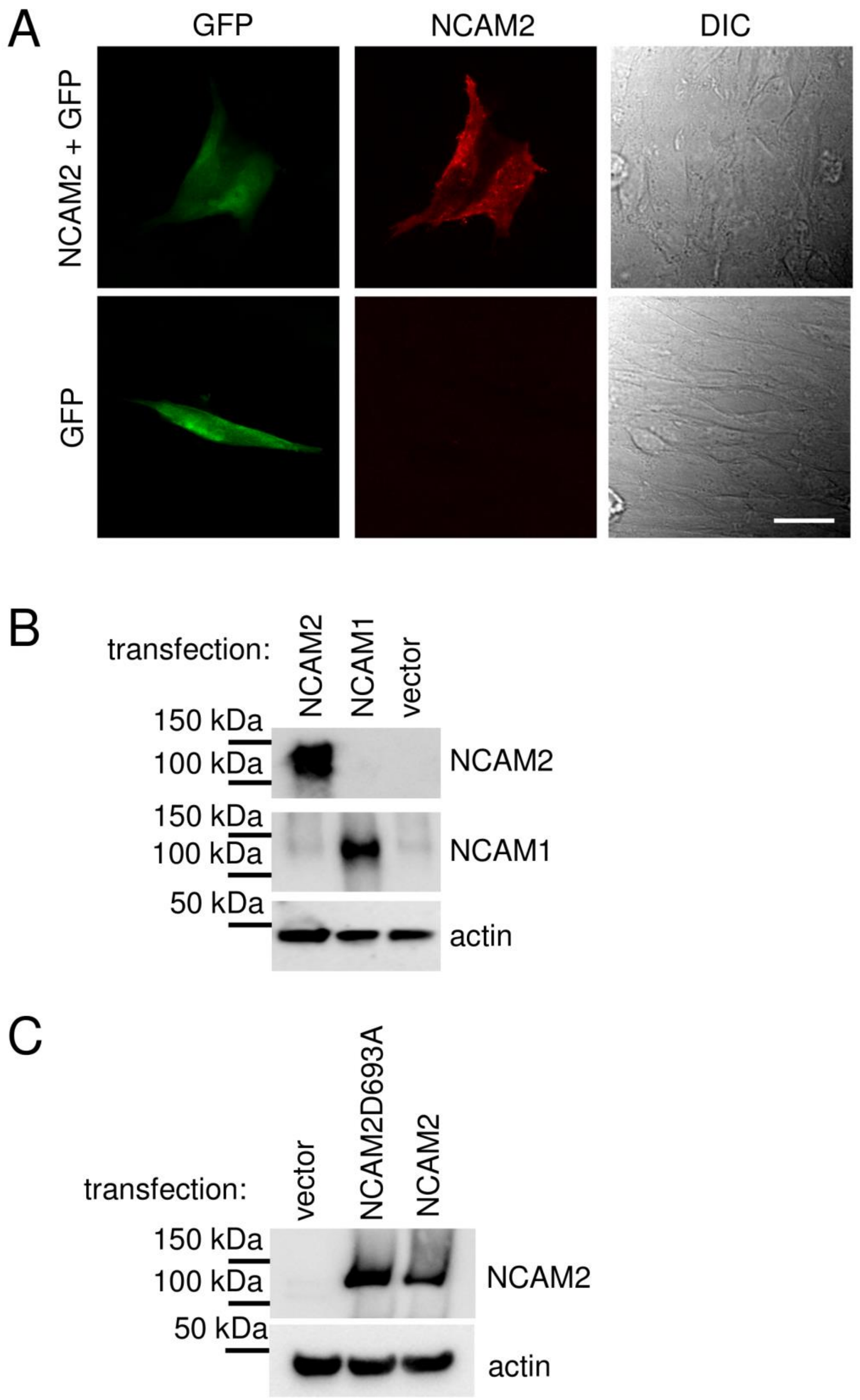

Supplementary Figure 2. NCAM2 antibodies specifically detect overexpressed NCAM2 in transfected CHO cells. **A** – CHO cells transfected with NCAM2 and GFP or with GFP alone were labeled with antibodies against NCAM2. Note NCAM2 labeling in co-transfected cells. Bar 20 μm. **B, C** – Western blot analysis of the lysates of CHO cells transfected with NCAM2, NCAM1 (NCAM140 isoform), or NCAM2D693A mutant. Note, that NCAM2 antibodies detect non-mutated NCAM2 and NCAM2D693A mutant, but not NCAM1. Actin served as loading control.

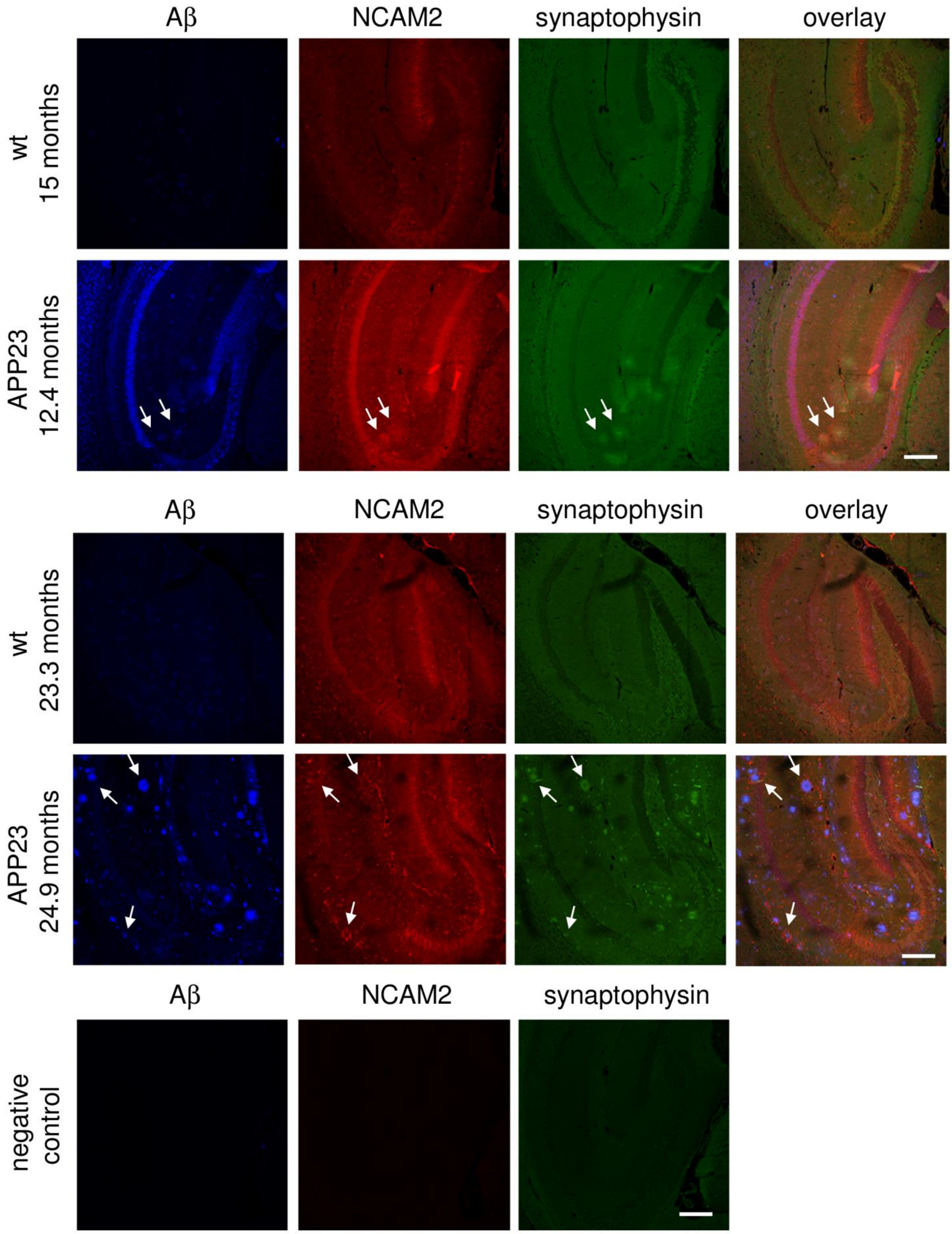

Supplementary Figure 3. NCAM2 co-localizes with plaques and Aβ aggregates in ageing APP23 mice.

Immunofluorescence images of the hippocampal sections of wild type and APP23 transgenic animals labelled with antibodies against Aβ, NCAM2 and synaptophysin are shown. Age of the animals is indicated on the left. In 12.4-months-old APP23 transgenic mouse, patches of increased NCAM2 and synaptophysin immunoreactivity co-localize with small Aβ aggregates (arrows). In 24.9-months-old APP23 transgenic

mouse, NCAM2 and synaptophysin immunoreactivity is found around Aβ positive plaques (examples shown with arrows). Omission of the primary antibodies eliminated the signal (negative control). Bar 200 μm.

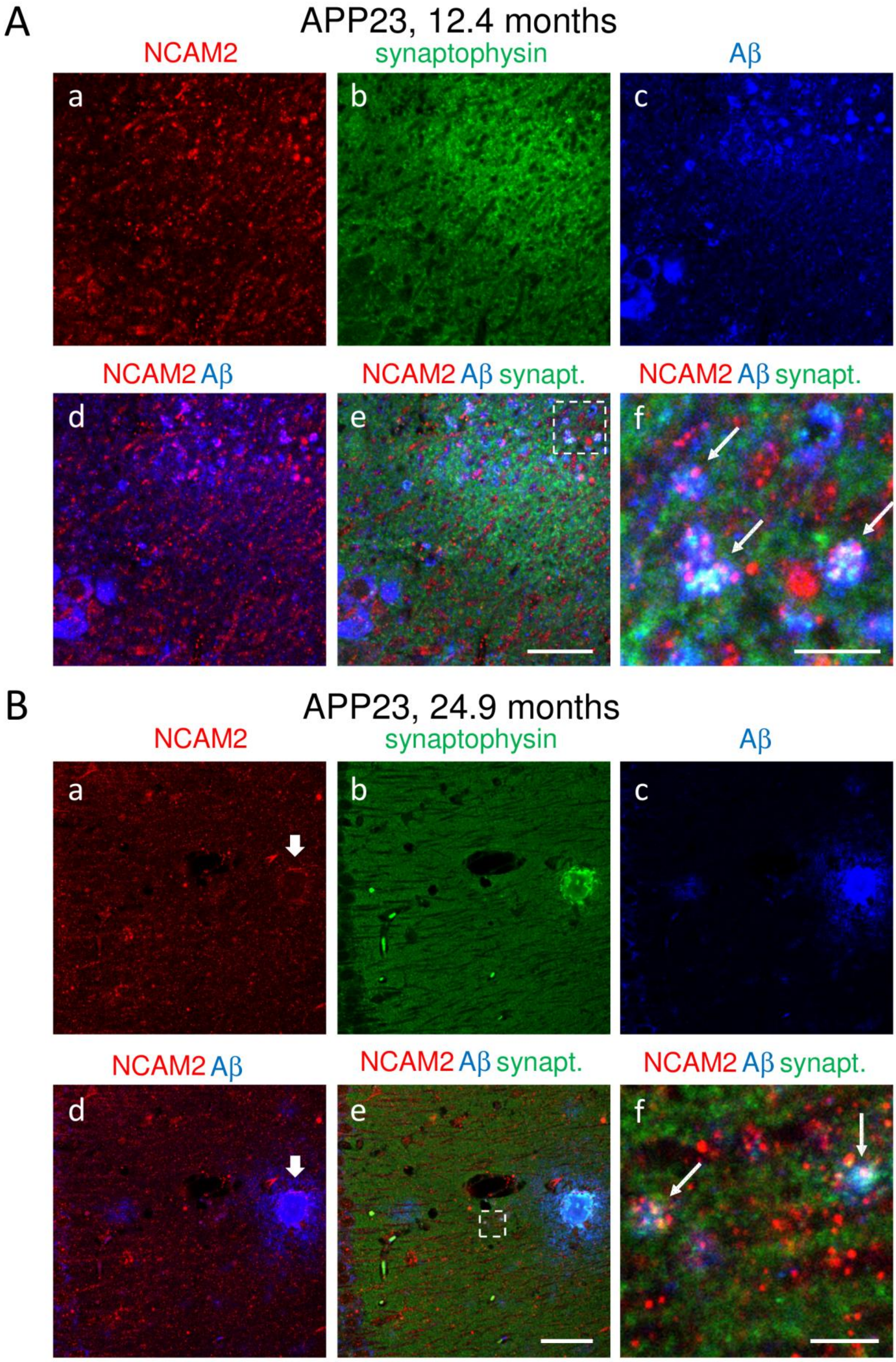

Supplementary Figure 4. NCAM2 co-localize with Aβ aggregates and is deposited around plaques.

A – Higher magnification of the CA2 region in the hippocampus of the 12.4-months-old APP23 transgenic mouse shown in Supplementary Fig. 3. Note accumulations of NCAM2 (**a**) and increased synaptophysin immunoreactivity (**b**) around Aβ aggregates (**c**) probably representing a nascent plaque.

Higher magnification of the area outlined in (**e)** is shown in (**f)**. Note NCAM2 accumulations co-localized with Aβ aggregates (arrows). Bar 20 μm (**a**-**e**), 5 μm (**f**).

B – Higher magnification of the Aβ positive plaque in the CA1 region of the hippocampus of the 24.9-months-old APP23 transgenic mouse shown in Supplementary Fig. 3. Note ring-like NCAM2 immunoreactivity (**a**, thick arrow) and increased synaptophysin labeling (**b**) around the plaque (**c**). Higher magnification of the area outlined in (**e)** is shown in (**f)**. Note NCAM2 accumulations co-localized with smaller Aβ aggregates (**f**, arrows). Bar 40 μm (**a**-**e**), 5 μm (**f**).

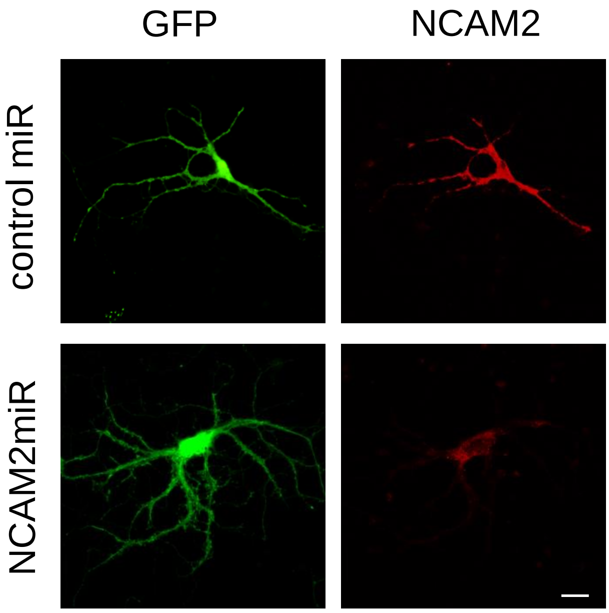

Supplementary Figure 5. Transfection with NCAM2 miR reduces levels of NCAM2 in cultured hippocampal neurons.

Representative images of cultured hippocampal neurons transfected with control miR or NCAM2 miR. Neurons were labeled for NCAM2. Note lower levels of NCAM2 in neurons transfected with NCAM2 miR. Bar 20 μm.

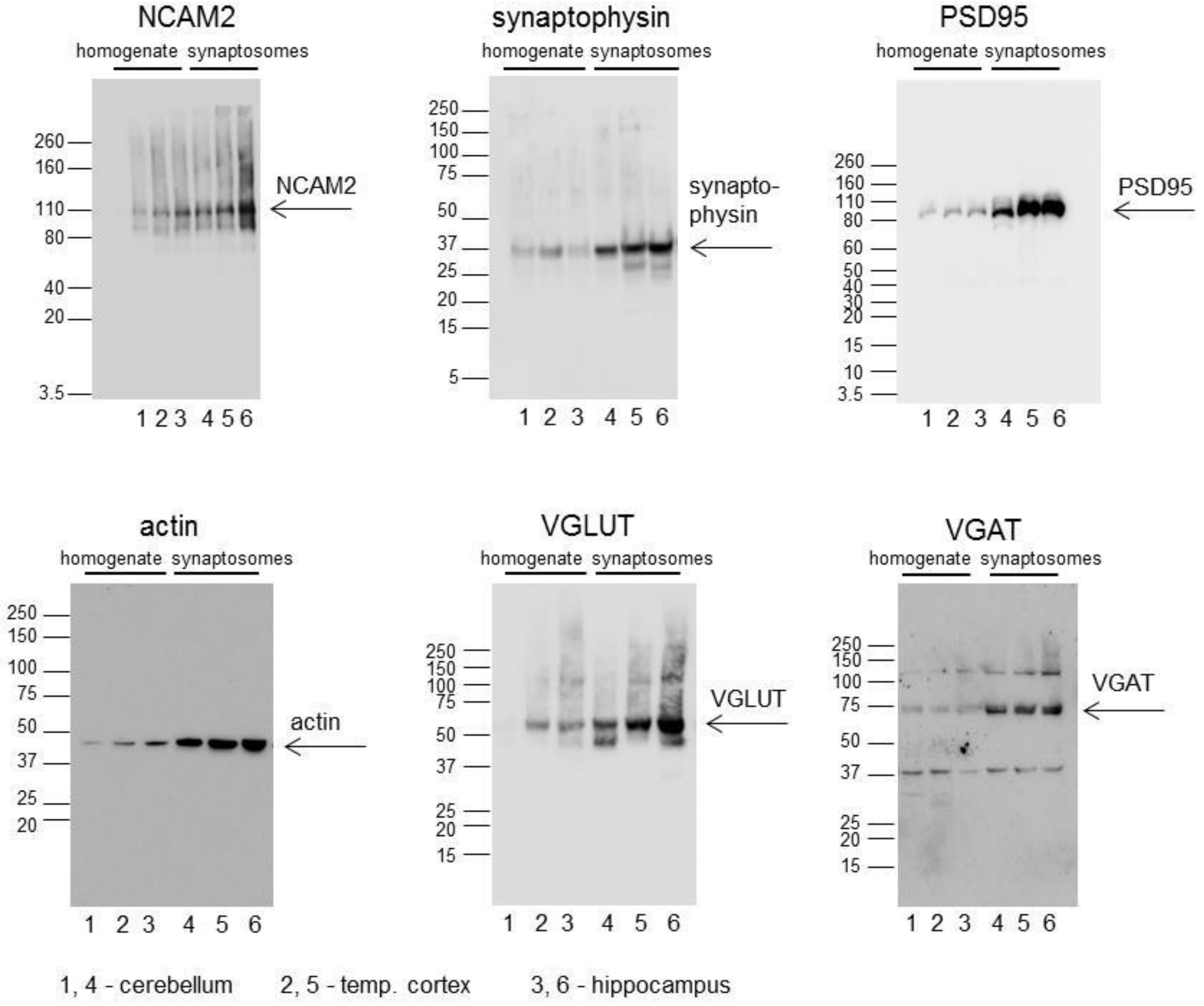

Supplementary Figure 6. Full length versions of the Western blots shown in Figure 1A (control) of the main paper.

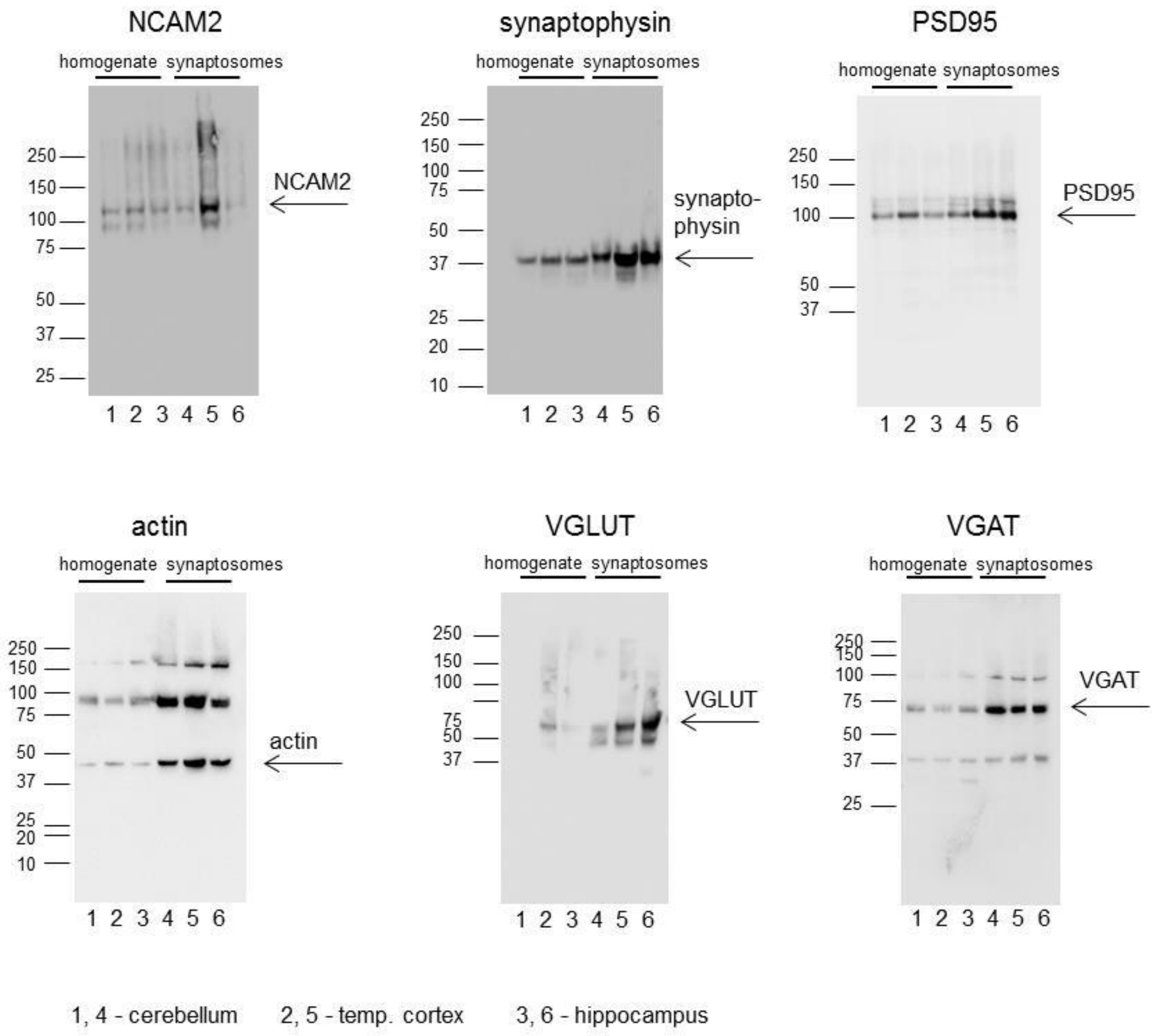

Supplementary Figure 7. Full length versions of the Western blots shown in Figure 1A (AD) of the main paper.

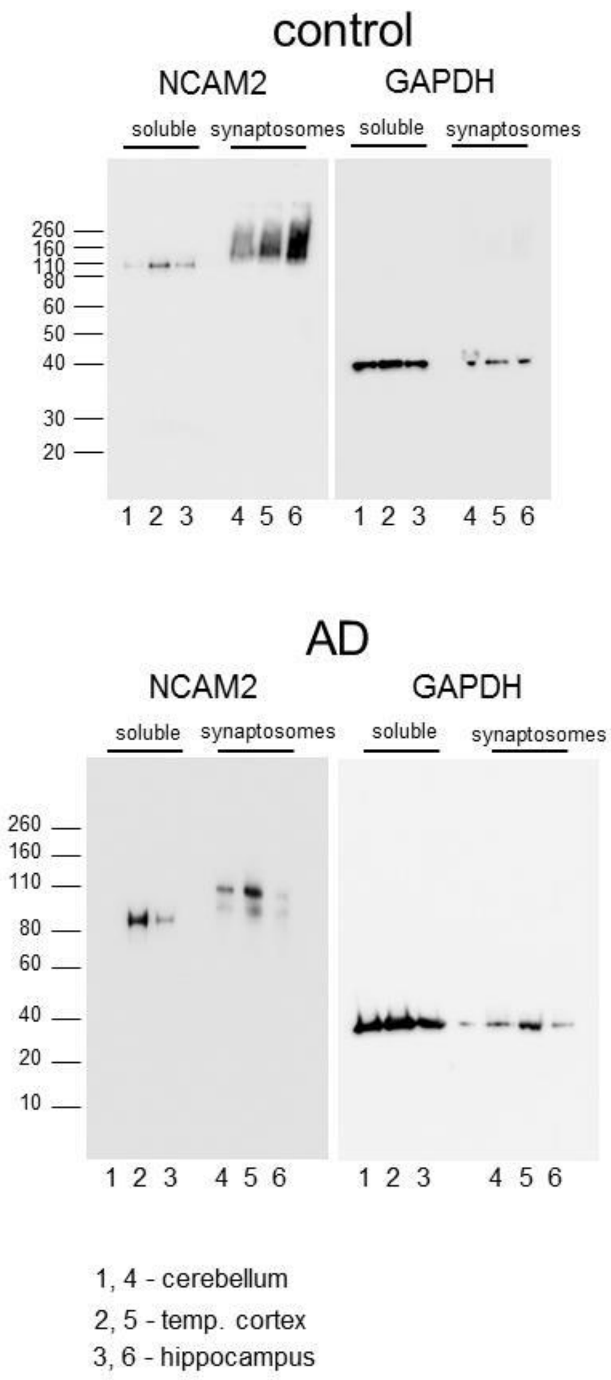

Supplementary Figure 8. Full length versions of the Western blots shown in Figure 1C of the main paper.

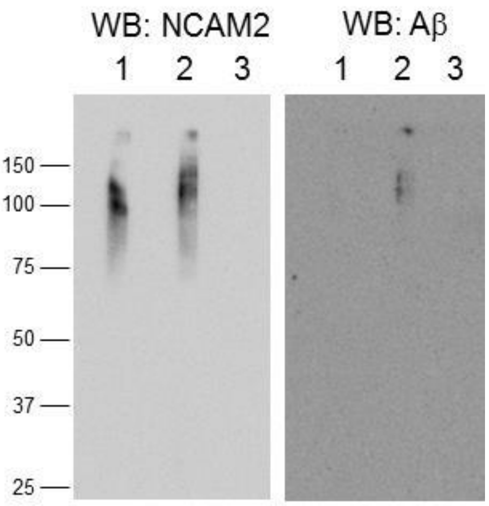

Supplementary Figure 9. Full length versions of the Western blots shown in Figure 3G of the main paper. To better separate the NCAM2 and NCAM2/Aβ bands in lanes 1 and 2, the Aβ oligomers applied in lane 3, which have the molecular weight of approx. 18 kDa (see Fig. 3E and G of the main paper), were allowed to run out from the gel.

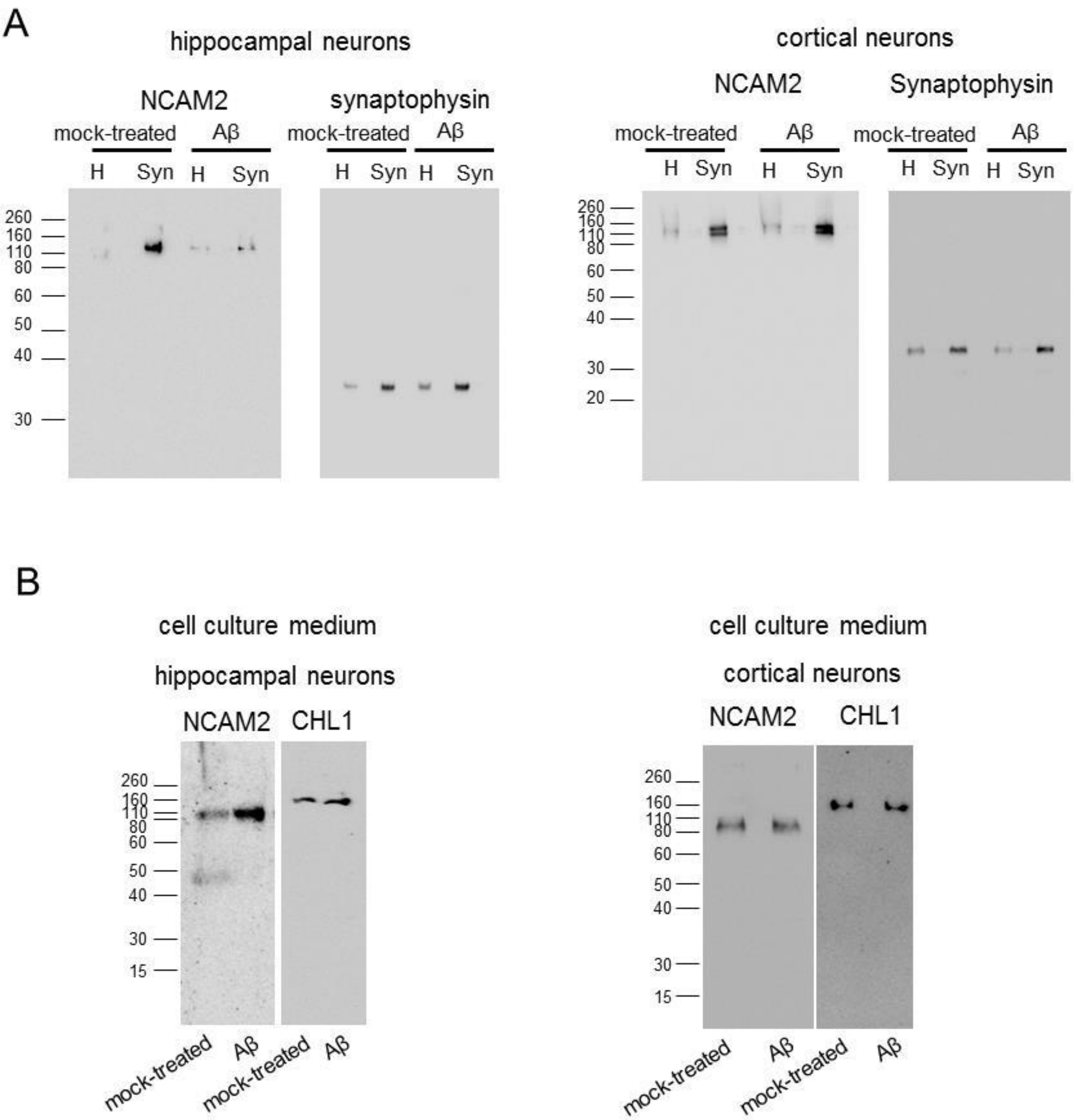

Supplementary Figure 10. Full length versions of the Western blots shown in Figure 6A (A) and Figure 6B (B) of the main paper.

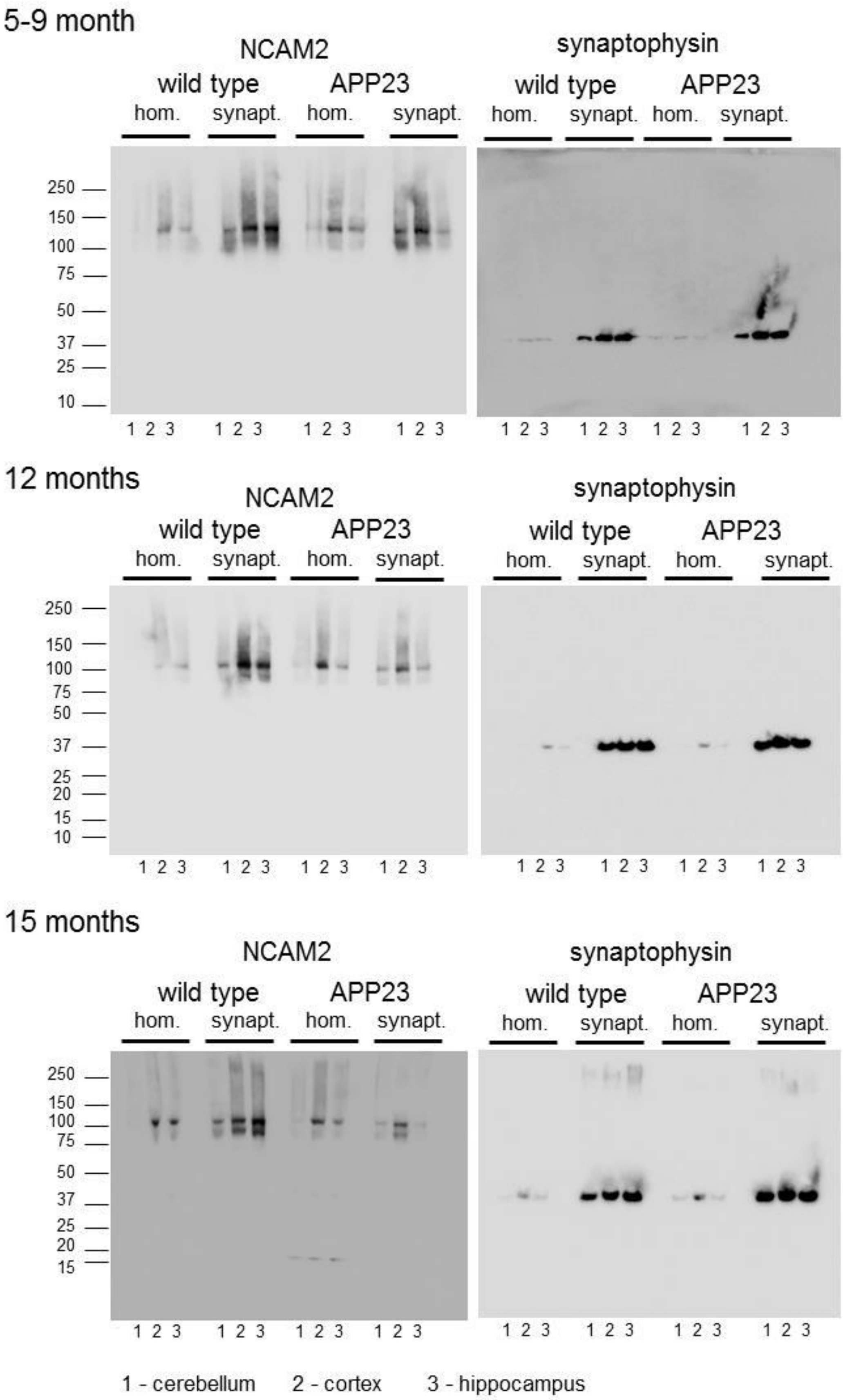

Supplementary Figure 11. Full length versions of the Western blots shown in Figure 8A of the main paper.

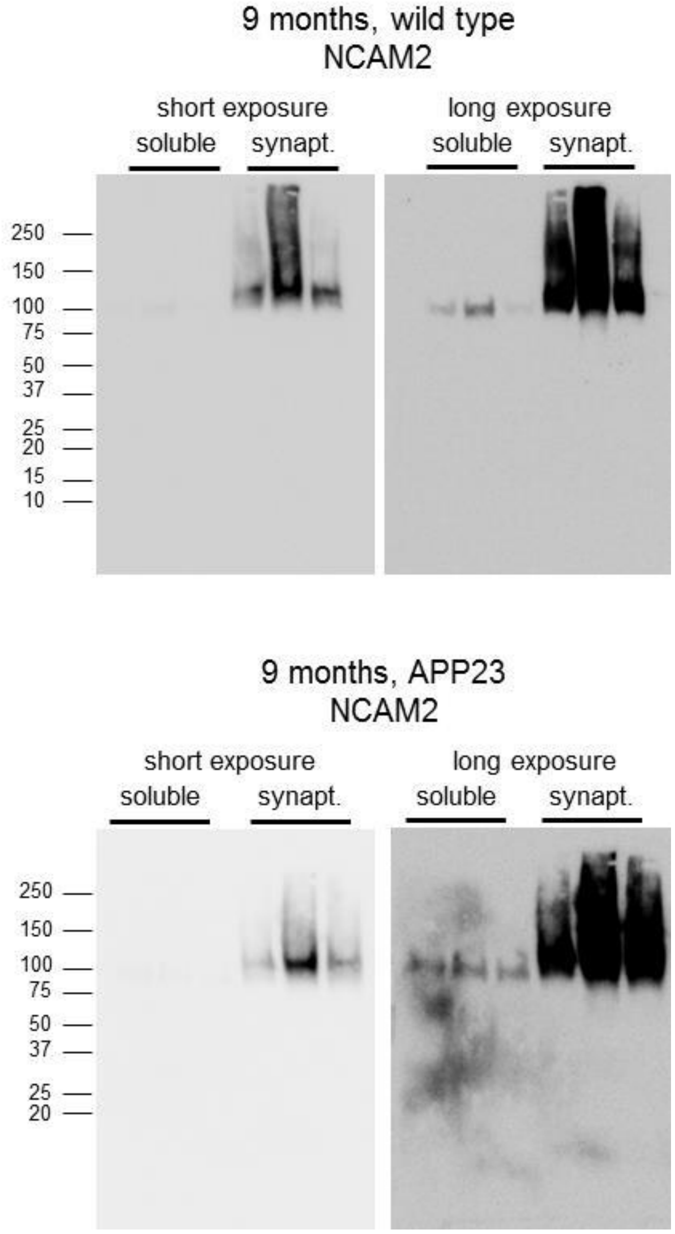

Supplementary Figure 12. Full length versions of the Western blots shown in Figure 8E of the main paper.

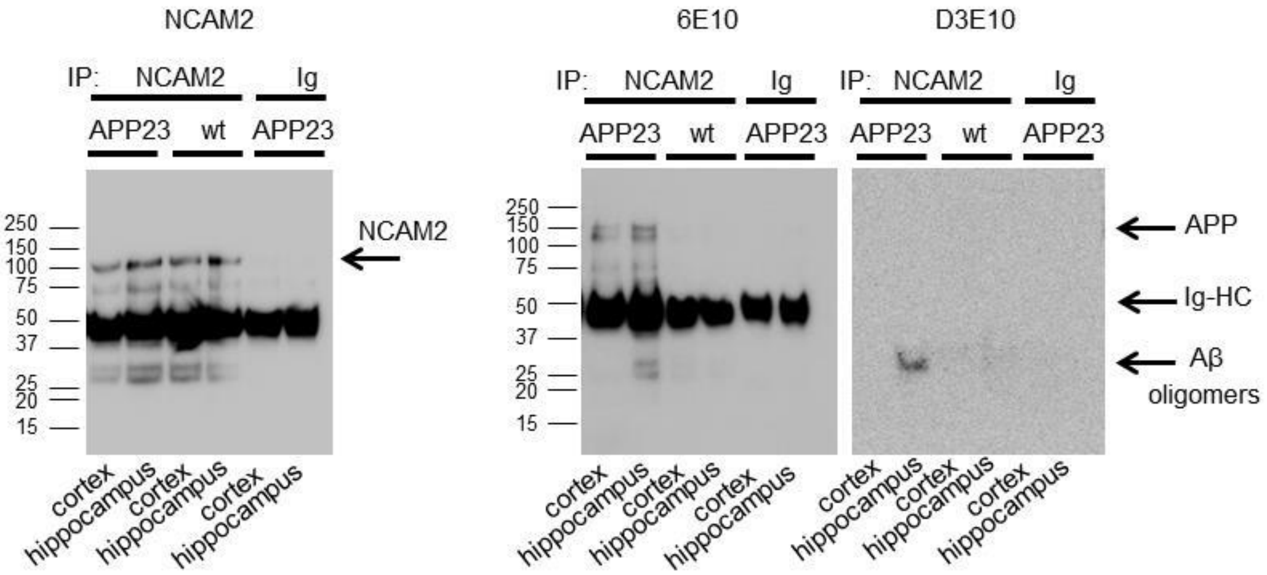

Supplementary Figure 13. Full length versions of the Western blots shown in Figure 8F of the main paper.

Supplementary Table 1. Case details for the human brain tissue analyzed in this study.

**Case**	**Age (years)**	**Gender**	**Control or AD (duration of the disease)**	**Braak stage (/6)**
1	81	Female	control	0
2	86	Male	control	1
3	69	Male	control	0
4	78	Female	control	0
5	69	Male	control	0
6	93	Female	control	0
7	85	Female	control	0
8	84	Female	control	3
9	102	Female	control	2
10	97	Female	control	2
11	94	Female	AD (1 year)	5
12	84	Female	AD (13 years)	6
13	83	Female	AD (7 years)	5
14	68	Male	AD (6 years)	6
15	69	Male	AD (5 years)	6
16	100	Female	AD (17 years)	6
17	76	Female	AD (11 years)	4
18	100	Female	AD (11 years)	5
19	80	Female	AD (10 years)	6
20	86	Male	AD (11 years)	6
